# Microbiological and Toxicological Hazards in Sewage Treatment Plant Bioaerosol and Dust

**DOI:** 10.3390/toxins13100691

**Published:** 2021-09-28

**Authors:** Justyna Szulc, Małgorzata Okrasa, Katarzyna Majchrzycka, Michael Sulyok, Adriana Nowak, Tomasz Ruman, Joanna Nizioł, Bogumiła Szponar, Beata Gutarowska

**Affiliations:** 1 Department of Environmental Biotechnology, Lodz University of Technology, 90-530 Łódź, Poland; justyna.szulc@p.lodz.pl (J.S.); adriana.nowak@p.lodz.pl (A.N.); beata.gutarowska@p.lodz.pl (B.G.); 2 Department of Personal Protective Equipment, Central Institute for Labour Protection—National Research Institute, 90-133 Łódź, Poland; kamaj@ciop.lodz.pl; 3 Institute for Bioanalytics and Agro-Metabolomics, Department of Agrobiotechnology University of Natural Resources and Life Sciences Vienna (BOKU), A-3430 Tulln, Austria; michael.sulyok@boku.ac.at; 4 Faculty of Chemistry, Rzeszów University of Technology, 35-959 Rzeszów, Poland; tomruman@prz.edu.pl (T.R.); jniziol@prz.edu.pl (J.N.); 5 Institute of Immunology and Experimental Therapy, Polish Academy of Sciences, 53-113 Wrocław, Poland; bogumila.szponar@hirszfeld.pl

**Keywords:** bioaerosol, harmful biological agents, metagenome analysis, mycotoxins, cytotoxicity, sewage treatment plant

## Abstract

Despite the awareness that work in the sewage treatment plant is associated with biological hazards, they have not been fully recognised so far. The research aims to comprehensively evaluate microbiological and toxicological hazards in the air and settled dust in workstations in a sewage treatment plant. The number of microorganisms in the air and settled dust was determined using the culture method and the diversity was evaluated using high-throughput sequencing. Endotoxin concentration was assessed with GC-MS (gas chromatography-mass spectrometry) while secondary metabolites with LC-MS/MS (liquid chromatography coupled to tandem mass spectrometry). Moreover, cytotoxicity of settled dust against a human lung epithelial lung cell line was determined with the MTT (3-(4,5-dimethylthiazol-2-yl)-2,5-diphenyltetrazolium bromide) assay and UHPLC-Q-ToF-UHRMS (ultra-high-performance liquid chromatography-quadrupole time-of-flight ultrahigh-resolution mass spectrometry) analysis was performed to determine the source of cytotoxicity. The total dust concentration in the sewage treatment plant was low and ranged from 0.030 mg m^−3^ to 0.044 mg m^−3^. The highest microbiological contamination was observed in sludge thickening building and screenings storage. Three secondary metabolites were detected in the air and sixteen in the settled dust. They were dominated by compounds typical of lichen and plants and *Aspergillus*, *Penicillium* and *Fusarium* genera mould. The settled dust from the sludge thickening building revealed high cytotoxicity to human lung epithelial cells A-549 (IC_50_ = 6.98 after 72 h). This effect can be attributed to a biocidal compound—didecyldimethylammonium chloride (DDAC-C10) and seven toxic compounds: 4-hydroxynonenal, carbofuran, cerulenin, diethylphosphate, fenpropimorph, naphthalene and onchidal. The presence of DDAC-C10 and other biocidal substances in the sewage treatment plant environment may bring negative results for biological sewage treatment and the natural environment in the future and contribute to microorganisms’ increasing antibiotics resistance. Therefore, the concentration of antibiotics, pesticides and disinfectants in sewage treatment plant workstations should be monitored.

## 1. Introduction

Over 300 km^3^ of sewage is produced globally every year, which, combined with the progressing urbanisation, leads to an increasing number of wastewater treatment plants (WWTPs) [1,2]. Wastewater treatment plants are complexes of technological buildings where industrial wastewater and sewage are treated, i.e., dissolved substances, colloids and suspensions are removed from wastewater before discharge to water or the ground [3]. The European Environment Agency (EEA) published a complete database of the existing urban WWTPs in Europe describing 25,906 such facilities [4]. In Poland, there are over 3257 WWTPs [3].

The wastewater treatment plants can be divided into a few different categories depending on the applied wastewater treatment methods and the related processes. In mechanical ones, only non-soluble contaminants (e.g., sedimented or floating solids and fats) are removed with grates, screens and grit chambers. In the chemical WWTPs, wastewater is treated through precipitation of some soluble compounds and neutralised with chemical methods (e.g., coagulation, sorption on active carbon). In biological WWTPs, organic contaminants and biogenic and refractory compounds are removed from wastewater through biological decomposition using microorganisms. Wastewater treatment plants with high removal of biogens enable higher nitrogen and phosphorus reduction [2,3]. Wastewater biological treatment systems include around a billion active microorganism species, and activated sludge can contain about 4 × 10^8^ cells mL^−1^. Wastewater biological treatment broadly uses aeration, mixing and compressing to intensify the growth and proliferation of the microorganisms. Wastewater turbulences, intensive flow rate and aeration are considered to promote bioaerosol formation and release into the wastewater treatment plant’s atmosphere [5,6].

The bioaerosol formed in the wastewater treatment plants includes numerous bacilli (*Klebsiella pneumoniae, Escherichia coli, Enterobacter agglomerans, Aeromonas hydrophila, Pseudomonas* spp.), haemolytic staphylococci (e.g., *Staphylococcus aureus*), haemolytic streptococci (*Streptococcus faecalis, Streptococcus pneumoniae*) and Gram-negative coccobaccilli such as *Acinetobacter*, some *Bacillus* genera and coliform bacteria [7,8]. Li et al. detected 300 bacteria species, including opportunistic pathogens such as *Comamonas testosteroni* and *Moraxella osloensis* in bioaerosol from a wastewater treatment plant [9]. Han et al. also point out the potential harmfulness of *Acinetobacter lwoffii*, *Aeromonas caviae*, *Arcobacter* spp., *Bacillus* spp., *Bacteroides* spp., *Flavobacterium* spp., *Mycobacterium* spp., *Pseudomonas fluorescens*, *Zoogloea* spp., *Kluyvera intermedia*, *Staphylococcus lentus*, *Clostridium* spp. and *Enterobacter* spp. bacteria to the health of wastewater treatment plant employees [10,11]. Wastewater and bioaerosol generated in a WWTPs were also reported as the source of drug-resistant microorganisms and genes that induce antibiotic resistance [12]. The dominant fungi identified in the wastewater treatment plant air included *Cephalotrichum* spp., *Alternaria* spp., *Penicillium* spp., *Monilia* spp. and *Aspergillus* spp. [13].

In addition to the potentially pathogenic microorganism species, wastewater treatment plant staff are exposed to inhalation of bacterial endotoxin from the Gram-negative bacteria cell wall. Smit et al. discovered a positive, dose-dependent relationship between exposure to endotoxins and adverse respiratory effects in humans, including wheezing breath, dyspnoea and cough [14].

The wastewater treatment plant staff often complain about ill-health, cough and breathing problems [15]. The most common diseases caused by exposure to harmful biological factors in wastewater include allergic alveolitis, rhinitis and pharyngitis, conjunctivitis, diarrhoea and other infections of the alimentary tract and central nervous system lesions [16]. Friis et al. discovered a higher number of stomach cancer cases among wastewater treatment plant staff, which can be related to the presence of *Helicobacter pylori* bacteria in the work environment [15].

A disease unit described as ‘Sewage Worker’s Syndrome’ is accompanied by such symptoms as general discomfort, weakness, acute rhinitis and fever [17].

The issue of harmful biological factors hazards in a WWTP’s work environment is known and broadly investigated. However, there are no comprehensive tests that would include the presence of cultivable and non-cultivable microorganisms and metabolites of microbiological origin in the air and settled dust in such facilities. No cytotoxicity analyses have been carried out so far for the dust in wastewater treatment plants.

That is why this paper was aimed to carry out a comprehensive evaluation of microbiological and toxicological hazards in a sewage treatment plant. It presents the quantity, diversity and profile of chemical compounds of biological origin (including endotoxins and secondary metabolites) occurring in the air and settled dust in a sewage treatment plant. The cytotoxicity was evaluated of settled dust from workstations in the sewage treatment plant against a human lung epithelial cell line and tests were carried out using UHPLC-Q-ToF-UHRMS (ultra-high-performance liquid chromatography-quadrupole time-of-flight ultrahigh-resolution mass spectrometry) to determine why dust from the sewage treatment plant was highly cytotoxic. Such evaluation can be used to define provisions to protect workers from risks to their health and safety, including the prevention of such risks arising or likely to arise from exposure to biological agents at workstations at WWTPs. The results can be utilised by health and safety professionals to recognise activities in which workers may be potentially exposed to biological agents due to their work and thus undertake preventive measures, including the use of appropriate personal protective equipment.

## 2. Results and Discussion

### 2.1. Microclimatic Conditions

The microclimate parameters at the workstations in the sewage treatment plant are summarised in Table 1. The air temperature in the tested places ranged from 17.4 °C (Workstation 2) to 26.4 °C (Workstation 6). The highest (73.1%) and the lowest (69.8%) relative air humidity were reported for the same workstations, respectively. The airflow rate ranged between 0.03 m s^−1^ (Workstation 4) and 0.87 m s^−1^ (Workstation 1).

The microclimate conditions at different workstations were highly diversified. Statistically, significant differences were revealed for the measurements carried out on consecutive days and for workstations located close to one another in an open space during measurements performed on the same day. In all cases, the relative humidity at the workstations was high (>57%), which can provide suitable conditions for microorganisms development in the air and their transmission to greater distances, considering the temperature of ca. 25 °C and the reported airflow rate values.

### 2.2. Dust Concentrations at Workstations

The total dust concentration in the sewage plant was very low and ranged from 0.030 mg m^−3^ (external background) to 0.044 mg m^−3^ (screenings storage; Table 2). At all measurement points, the aerodynamic diameter of the dominant suspended dust fraction was less than 1 µm (Table 2). Its share in the total quantity of the measured dust ranged from 73.9% in the sludge thickening building to 92.3% in the sewage treatment plant’s environment (screenings storage). The particles with diameters ranging from 1 to 4 µm (1.7–7.5%) constituted the smallest part of the total dust amount.

The dust concentration values do not exceed the permissible threshold values at the workstations, and so according to domestic law, there is no need to use respiratory protection against particles. The use of respiratory protection is then voluntary, and the adequate equipment is selected depending on the user’s work comfort needs. The possible exposure to the SARS-COV-2 virus is also taken into account.

### 2.3. Microbial Contamination

The bacteria count in sewage treatment plant air ranged from 3.05 × 10^2^ CFU m^−3^ (Workstation 3) to 3.71 × 10^3^ CFU m^−3^ (Workstation 4). Actinomycetes were detected only at Workstation 2 (15.0 CFU m^−3^) and the same number was detected in the control atmospheric air (0.5 km away from the sewage treatment plant). The count of mannitol-positive Staphylococci ranged from 2.5 CFU m^−3^ to 65.0 CFU m^−3^. The concentration of *Enterobacteriaceae* family bacteria ranged from 2.5 CFU m^−3^ (Workstation 1) to 1.98 × 10^2^ CFU m^−3^ (Workstation 5) (Table 3). The count of *Pseudomonas fluorescens* bacteria was also low and amounted to 7.5 CFU m^−3^–40.0 CFU m^−3^. Haemolytic *Staphylococci* were most numerous at Workstation 5 (3.25 × 10^2^ CFU m^−3^), while their lowest counts were observed at Workstation 3 and 5 (50.0 CFU m^−3^). The fungi count at the tested workstations ranged from 1.91 × 10^3^ CFU m^−3^ (Workstation 4) to 3.23 × 10^4^ CFU m^−3^ (Workstation 5), while for xerophilic fungi, the values ranged from 1.13 × 10^3^ CFU m^−3^ (Workstation 3) to 2.39 × 10^4^ CFU m^−3^ (Workstation 6) (Table 3).

The results show that the highest microbiological contamination occurred at Workstation 4 (sludge thickening building) and 5 (screenings storage). However, a statistical analysis of the results comparing the microbiological contamination in the air from the tested workstations and control atmospheric air revealed only a few statistically significant differences. It suggests a comparable microbiological condition of the air in and outside the sewage treatment plant premises.

The microorganisms count in the sewage treatment plant air was tested in many previous papers, but the characteristic levels have not been determined, so there is still a need to conduct microbiological analyses in such facilities. Li et al. detected the highest count of bacteria (1.7 × 10^3^ CFU m^−3^) and fungi (9.3 × 10^2^ CFU m^−3^) in a sewage treatment plant in China at sludge thickening stations [9]. The microorganisms count reported by other authors for sewage treatment plant air is highly diversified and ranges from 10^2^ to 10^5^ CFU m^−3^ [2,8]. Moreover, bacteria concentrations in bioaerosol in such work environments can be twice higher and fungi concentrations 8–10 times higher in spring than winter [13]. The volume of microorganisms emission to the air in a sewage treatment plant was proven to vary and depend on the season but also the facility size, quantity and nature of the treated sewage and the sewage treatment process, i.e., sewage treatment plant operation and applied technology [2,18].

Settled dust at the tested sewage treatment plant workstations occurred only in the sludge thickening building, while other workstations were located in an open space, where settled dust did not accumulate.

**Figure 1:**
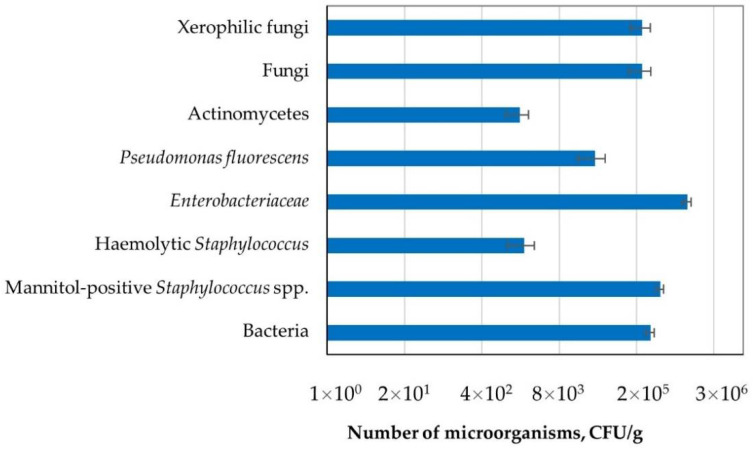
The number of microorganisms in the dust collected from the sludge thickening building in the sewage treatment plant.

*Enterobacteriaceae* family bacteria (1.14 × 10^6^ CFU g^−1^) and mannitol-positive Staphylococci (4.03 × 10^5^ CFU g^−1^) dominated the dust samples collected from the sludge thickening building, while the total bacteria count was 2.73 × 10^5^ CFU g^−1^. A similar contamination level was reported for fungi and xerophilic fungi (2.00 × 10^5^ CFU g^−1^). In the tested dust, Actinomycetes (1.74 × 10^3^ CFU g^−1^) and haemolytic Staphylococci (2.08 × 10^3^ CFU g^−1^) (Figure 1) were the least numerous.

The literature provides no data on the microorganisms count in the settled dust from workstations in a sewage treatment plant. Only settled dust from the agricultural work environment (poultry breeding rooms, cereal dust) was microbiologically characterised. A higher count of bacteria (3.33 × 10^7^ CFU g^−1^–1.57 × 10^8^ CFU g^−1^) and *P. fluorescens* bacteria (1.00 × 10^5^ CFU g^−1^–5.73 × 10^5^ CFU g^−1^) was reported. The haemolytic Staphylococci count was higher for poultry farm dust (8.50 × 10^5^ CFU g^−1^) and similar for cereal dust (2.98 × 10^3^ CFU g^−1^) [19,20]. The counts of Actinomycetes (≤2.05 × 10^3^ CFU g^−1^) and xerophilic fungi (3.35 × 10^5^ CFU g^−1^–4.45 × 10^5^ CFU g^−1^) were on a similar level.

The lowest counts were in turn reported for mannitol-positive Staphylococci (1.20 × 10^4^ CFU g^−1^–3.49 × 10^4^ CFU g^−1^), *Enterobacteriaceae* family bacteria (≤2.30 × 10^4^ CFU g^−1^) and fungi (7.48 × 10^4^ CFU g^−1^–8.75 × 10^5^ CFU g^−1^).

### 2.4. Diversity of Microorganisms from the Sewage Plant Workstations

**Figure 2:**
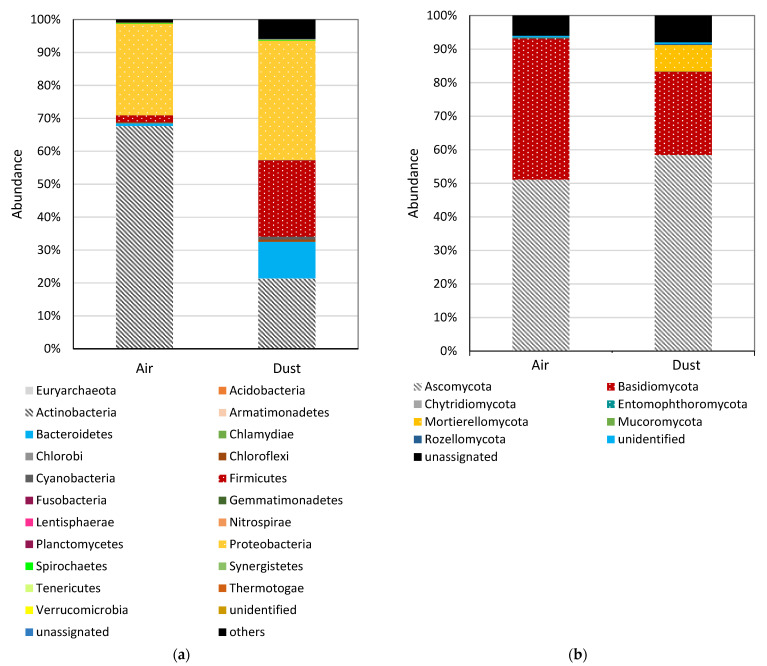
Phylogenetic distribution of (**a**) bacteria and (**b**) fungi sequences assigned to the genera in the dust and air samples.

High-throughput sequencing of the DNA from the air and settled dust collected at Workstation 4 in the sewage treatment plant (sludge thickening building) revealed a high phylogenetic diversity of the microorganisms (Figure 2a,b). One hundred and seventy (170) genera of bacteria/archeons and 475 genera of fungi, representing 19 and 9 types, respectively, were detected in the dust. The number of bacteria/archeons genera identified in the air amounted to 166 (19 types), and the number of fungal genera amounted to 475 (7 types).

Both the dust and the air were mainly contaminated by Actinobacteria (68.0 and 21.0%), Proteobacteria (28.0 and 36.0%) and Firmicutes (2.0 and 23.0%). Moreover, a significant share was discovered of the bacteria genus and Bacteroidetes (11.0%) in the settled dust in the sewage treatment plant. Other bacteria types constituted less than 1% OTU (operational taxonomic unit) in the tested air and settled dust (Figure 2a).

The bacteria genera observed most often among Actinobacteria in the dust included *Bifidobacterium* (5.0%), *Gordonia* (3.0%), *Corynebacterium* (1.0%), *Terracoccus* (1.0%), *Candidatus Microthrix* (0.94%) and *Cellulosimicrobium* (0.71%). The Proteobacteria were dominated by *Sphingomonas* (4.0%), *Acinetobacter* (4.0%), *Psychrobacter* (4.0%), *Pseudomonas* (4.0%), *Serratia* (2.0%) and *Stenotrophomonas* (1.04%) genera. The Firmicutes identified most often in the sewage treatment plant’s dust include *Turicibacter* (1.0%). The *Chryseobacterium* (10.0%) genus representing Bacteroidetes occurred in the dust with high OTU values.

The air collected from the sewage treatment plant was dominated by such Actinobacteria genera as *Cellulosimicrobium* (59.0%) and *Bifidobacterium* (6.0%). *Pseudomonas* (12.0%) and *Stenotrophomonas* (8.0%) were the most common Proteobacteria. The presence of 44 bacteria genera with the OTU < 1% (Table S1) was confirmed among the Firmicutes.

Ascomycota and Basidiomycota were the most common fungi in both sample types (dust and air) (Figure 2b). In the settled dust, *Mycosphaerella* (12.0%), *Cladosporium* (6.0%), *Neoascochyta* (5.0%), *Alternaria* (4.0%), *Didymella* (4.0%), *Chalastospora* (3.0%), *Botrytis* (2.0%) and *Citeromyces* (2.0%) had the highest OTU among Ascomycota (59.0%). Basidiomycota were dominated by *Cryptococcus* (3.0%), *Cystobasidium* (3.0%), *Rhodotorula* (1.0%) and *Sporobolomyces* (1.0%). The *Aspergillus* and *Penicillium* genera fungi quantities were similar (2.0–3.0%) in the dust and air samples. Moreover, a high percentage of *Mortierella* genera representing *Mortierellomycota* was observed in the dust. 

For fungi in the air samples, the most common DNA sequences related to Ascomycota included *Cladosporium* (14.0%), *Mycosphaerella* (7.0%), *Alternaria* (5.0%), *Didymella* (2.0%), *Candida* (2.0%) and *Dissoconium* (1.0%) genera. The highest OUT of Basidiomycota in the air was identified for *Sporobolomyces* (4.0%), *Hypholoma* (3.0%), *Udeniomyces* (3.0%), *Coprinellus* (1.0%), *Bjerkandera* (1.0%) and *Malassezia* (1.0%) (Table S2).

Tests presenting in such a detailed way the bacteria and fungi diversity in the sewage treatment plant’s air and settled dust were carried for the first time under this study. Previously, many authors were interested in the qualitative and quantitative composition of microorganisms in sludge and sewage. 

The bacteria genera most often isolated from the air in mechanical and biological sewage treatment plants included *Citobacter, Enterobacter, Klebsiella, Serratia* and *Pantoea*, whereas pathogenic bacteria such as *Salmonella, Escherichia* and *Shigella* were rarely isolated [21,22]. *Absidia, Actinomucor, Alternaria, Aspergillus, Cladosporium, Fusarium, Geotrichum, Mucor* and *Penicillium* mould and *Candida, Cryptococcus* and *Rhodotorula* yeast were most often described as fungi characteristic for sewage treatment plant environment [21,23].

In the present study, a high-throughput DNA sequencing from the samples collected in the sludge thickening building revealed a broader spectrum of microorganisms occurring in the sewage treatment plant air and dust, including potentially pathogenic microorganisms that can threaten human life. The presence of *Corynebacterium* and *Pseudomonas* genera was detected (12.0%)—their pathogenic species represent Hazard category 2 according to Directive 2000/54/EC of the European Parliament and of the Council of 18 September 2000 on the Protection of Workers from Risks Related to Exposure to Biological Agents at Work. The identified fungi with high OTU values included *Aspergillus*, *Candida*, *Cryptococcus* and *Penicillium*, which may include species representing Employee’s Health Hazard Group 2. 

Moreover, *Candidatus Microthrix* bacteria representing filiform bacteria often occurring in the air in sewage treatment plants using activated sludge technology were detected in the tested samples. The presence of such bacteria can be related to sludge sedimentation problems, i.e., “swelling” [24,25].

### 2.5. Endotoxin Concentrations

**Figure 3:**
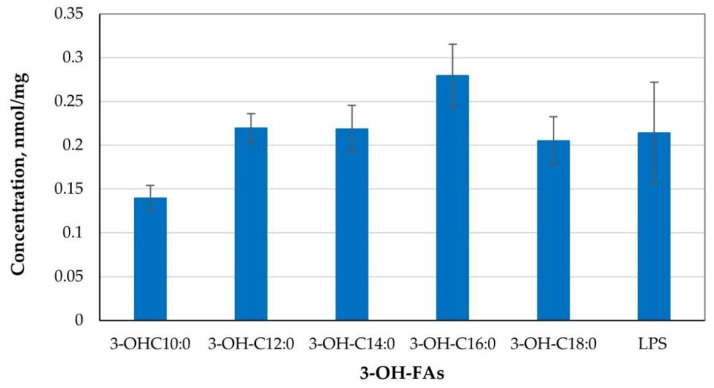
Bacterial endotoxin concentration in settled dust from the sewage treatment plant.

Settled dust in the tested sewage treatment station occurred only in the sludge thickening building (Workstation 4). The endotoxin concentration in the dust samples amounted to 0.214 nmol LPS mg^−1^ on average (Figure 3).

The concentration was lower than that published in the data for dust from livestock breeding facilities. Pomorska et al. documented the LPS concentration in the livestock buildings for horses at 11 nmol LPS mg^−1^ to 110 nmol LPS mg^−1^; in the livestock buildings for poultry, it ranged from 65 nmol LPS mg^−1^ to 176 nmol LPS mg^−1^ and for sheep from 61 nmol LPS mg^−1^ to 226 nmol LPS mg^−1^ [26]. The bacterial endotoxin concentrations obtained in the present study are higher than the values described in the literature for household rooms of different functions (kitchen, bedroom, living room—from 0.092 nmol LPS mg^−1^ to 0.155 nmol LPS mg^−1^) [27]. Based on studies carried out in many sewage treatment plants in Finland and Norway, the endotoxin concentration in such work environments is diversified and ranges from 0.6 to 370 ng m^−3^ in the air [28,29]. The highest endotoxin concentrations were confirmed for the sewage sludge treatment areas.

Interestingly, endotoxins from different bacteria can differ in their molecular structures [30,31]. Moreover, bacteria can adopt to an unfavourable environment to ensure viability. It is known that bacteria are able to modify their primary LPS structure under certain growth conditions. Thereby they can reinforce the external membrane to assure optimal protection against the environment [32,33]. 

The literature examples demonstrate that exposure to bacterial endotoxin through inhalation may result in respiratory tract inflammation and toxic pneumonia caused by the non-specific activation of alveolar macrophages that release inflammation mediators. Endotoxins may also cause fever, chills, cough and flu symptoms [34,35].

### 2.6. Secondary Metabolites

In the sludge thickening building of the sewage treatment plant, three chemical compounds were detected in the air and 16 in the settled dust (Table 4). Metabolites characteristic for *Aspergillus* (3-Nitropropionic acid) and *Penicillium* (Flavoglaucin) genera mould were detected in the air, next to metabolites not related to any specific mould genus (non-specific)—asperphenamate. Compounds produced by *Penicillium* spp., (quinolactacin A, citreohybridinol, flavoglaucin, pentoxifylline), *Fusarium* spp. (beauvericin, enniatin A1, enniatin B, enniatin B1) and *Aspergillus* (3-Nitropropionic acid) dominated in the air. Lichen (lecanoric acid, usnic acid) and plant (prunasin) metabolites, and non-specific compounds (asperglaucide, asperphenamate, cyclo(L-Pro-L-Tyr, emodin) (Table 4) were also detected. It is worth pointing out that the concentrations of lichen and plant metabolites were higher (14–123 ng g^−1^) than those of metabolites of microbiological origin (concentration range: 0.24–18.1 ng g^−1^).

Previously, the literature published no such detailed analysis of mycotoxins content in the air and settled dust from a sewage treatment plant. The latest studies included assessing four metabolite concentrations: aflatoxin B1, gliotoxin, ochratoxin A and sterigmatocystin. The authors detected in the air only aflatoxin B1 and sterigmatoxin at concentrations lower than ng m^−3^ [36].

### 2.7. Cytotoxic Effects of Dust Samples

**Figure 4:**
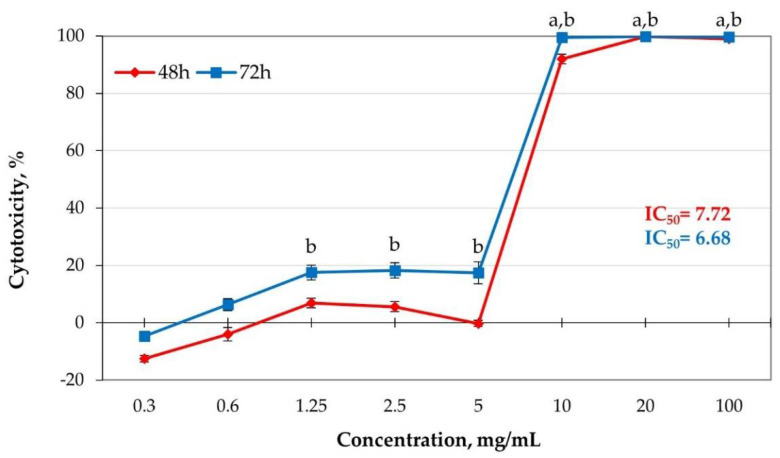
Curve presenting cytotoxic activity of water-soluble dust sample fractions towards A-549 cells. Each point stands for the mean absorbance values from four repetitions (±SD). Results statistically different from negative control: a after 48 h; b after 72 h exposition (*p* < 0.05, ANOVA).

This study is the first to show the cytotoxic effect of the settled dust samples from a sewage treatment plant. Human lung epithelial cells A-549 (Photo 1) were exposed to 0.3 to 100 mg mL^−1^ concentrations of water-soluble fractions of dust for 48 and 72 h. The curve representing the test dust’s cytotoxicity is shown in Figure 4.

High cytotoxicity of the tested dust was observed. Concentrations of 20 mg mL^−1^ and more caused 100% cytotoxicity for the tested cells after 48 h and 72 h of exposure. Low IC_50_ values (6.98–7.72%, depending on the exposure duration) confirm the high cytotoxicity of the tested dust, which inhibits the test cell population growth by 50%. High cytotoxicity of the dust settled in the sludge thickening buildings suggests the presence of non-biological inhalation hazards at the tested workstation, related to the process carried out at the workstation. 

**Figure 5:**
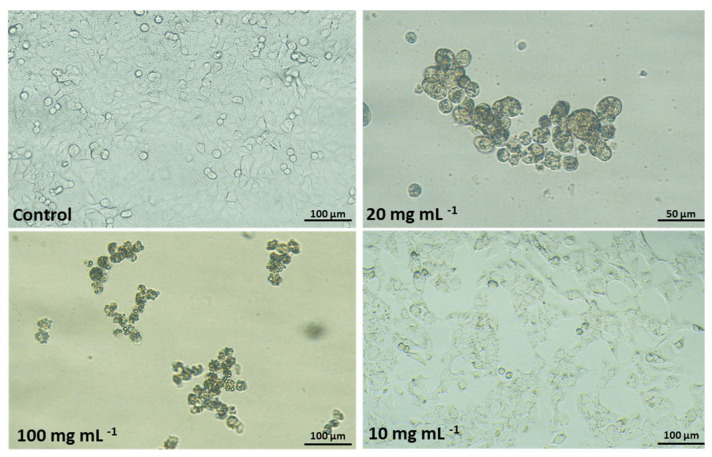
Morphology of human lung cells A-549 in culture after 48-h exposition to dust sample. Objectives 20 and 40× (Nikon Ts2, contrast EMBOSS, Japan).

Changes in both cell culture and morphology were also observed (Figure 5).

Control cells formed a regular monolayer with typical homogenous rhomboid cells and a clearly outlined cell nucleus and cytoplasm. After exposition to dust samples, a significant decrease in A-549 cells density, destruction of monolayer (no confluence), increased the amount of detached cells, and floating cells were detected especially in the presence of two highest tested concentrations (20 and 100 mg mL^−1^). Vacuolisation of the cell cytoplasm, intracytoplasmic granules and chromatin condensation were typical for lower concentration (10 mg mL^−1^). To conclude, the main mechanism of cytotoxic action was monolayer reduction—thus the high cytotoxicity. High cytotoxicity of dust samples may be caused by the presence of fungi metabolites, such as beauvericin and enniatins, which are produced by *Fusarium* species. Several authors demonstrated cytotoxic activity of beauvericin. It can induce apoptosis in A-549 cells through cell cycle arrest in the S phase and apoptosis through the MEK1/2/ERK42/44/90RSK signalling pathway [37]. Beauvericin showed cytotoxicity in MTT assay against A549 cells with IC_50_ value of 10.4 μM [37]. Lee et al. investigated the cytotoxic activity of beauvericin and enniatins using the sulphorhodamine B (SRB) method [38]. They observed very strong cytotoxicity of these compounds. The EC_50_ values (the concentration of a drug that gives a half-maximal response) were as follows: from 0.5 to 1.54 μM for enniatins and 1.43 μM for beauvericin.

In our research, the metabolites of *Aspergillus, Penicillium* and *Fusarium* species dominated the air and dust samples. It is worth noting that according to our best knowledge, the impact of sewage treatment plant dust on human lung cells has not been published so far. The literature shows the effect of individual metabolites of microbiological origin, e.g., mycotoxins, or antibiotics and other substances, but so far the complex mixture of dust from the working environment in a sewage treatment plant has not been studied, the total impact of which may be different. According to the International Agency for Research on Cancer (IARC), some mycotoxins, depending on the mould species, are classified carcinogenic to humans, but they were not detected in our study [39]. Instead, naphthalene was present, classified as ‘possibly carcinogenic to humans’ (Group 2B) [39]. It also could contribute to the strong cytotoxicity of our samples. Naphthalene is an air pollutant due to emissions from the chemical industry. It is used to synthesise various chemical compounds, dyes, insecticides, solvents, synthetic resins and tannins [40]. Its presence may indicate the origin of wastewater from industrial sources. The high cytotoxicity of the dust settled in the sludge thickening building indicates the presence of non-biological inhalation hazards at this workplace, related to the technological process carried out.

### 2.8. Targeted and Untargeted UPLC-HRMS Analysis

The results obtained in the process of identification of toxic compounds in dust are summarised in Table 5. The targeted identification of pesticides was performed using the TargetScreener (Bruker Daltonics) method based on tight tolerances matching of analyte retention time, precursor *m*/*z*, fragments *m*/*z*, precursor and fragments isotopic pattern and also precursor-to-fragment signals intensity ratios.

TargetScreener broadband CID (bbCID) method in positive mode confirmed the presence of didecyldimethylammonium chloride (DDAC-C10; Figure S1). DDAC-C10 is an alkyl quaternary ammonium salt with chain lengths of ten carbons widely used in numerous products for its broad bactericidal, viricidal and fungicidal properties. The broad toxic action of this compound on cells is based on its strong binding to negatively charged lipid bilayers leading to dissociation of the cell-membrane components and, as a result to cell death. DDAC-C10 is widely used, for example, as a biocide for the cleaning/sanitation of surfaces in the areas of food, feed and medical production sectors, and domestically, as a pesticide in ornamental plants protection products, as an antiseptic for wood and as a fungicide for coolants [41]. During the COVID-19 pandemic, large quantities of disinfectants (Penetone XF-7117, Maquat 256-MN, Vesta-Syde SQ 64 Ready-ToUse Disinfectant, Multi-Purpose Disinfectant 10 Cleaner, FSC 35K and many others) containing such active substances as DDAC-C10 have been recommended for the control of the virus SARS-CoV-2 by Environmental Protection Agency (EPA). These disinfectants have been commonly used in households, public buildings and workplaces [42]. The compound in the dust in the sewage treatment plant may originate from two sources: tested workstation after disinfection or sewage. The presence of DDAC in the wastewater treatment plant environment may affect the emergence of drug-resistant microorganisms. Bacterial resistance to quaternary ammonium compounds (QAC) including DDAC is among the best-studied of disinfectant resistance mechanisms. Most QAC-resistant isolates (Gram-positive and Gram-negative bacteria) are resistant by virtue of possessing one or more efflux pumps [43,44]. These pumps are membrane-bound, proton-motive force-dependent cation export proteins that belong either to the major facilitator family of transport proteins or to the small multidrug resistance protein family.

When resistance to one antibacterial agent is accompanied by the appearance of resistance to another agent can be the result of cross-resistance or co-resistance [45]. Cross-resistance can occur when different antimicrobial agents attack the same target, initiate a common pathway to cell death, or share a common route of access to their respective targets. Co-resistance can occur when the genes specifying resistant phenotypes are located together on a mobile genetic element (e.g., plasmid or transposon). Cross-resistance with other classes of antimicrobial agents via alteration of the efflux pump cannot be ruled out, particularly for cationic compounds like the aminoglycosides. Likewise, coresistance of disinfectant-resistant bacteria to antibiotics occurs as a result of QAC resistance genes being located on transmissible plasmids and within conserved regions of integrons, each of which has been shown to carry multiple antibiotic resistance genes [45]. Plasmids from a number of clinical isolates bearing qac genes and demonstrating a range of resistance phenotypes to a variety of antibiotics were characterised in literature [46]. Lemaitre et al., found plasmids bearing genes for QAC and tetracycline resistance e.g., for Listeria spp [47].

Additional AutoMSMS measurements in positive and negative ionisation modes were carried out and analysed in MetaboScape 2021 software for untargeted data profiling. Annotation was based on automatic matching of precursor *m*/*z* up to 2 ppm error, isotopic pattern matching up to 15 mSigma, and MSMS comparison. Seven toxic compounds were identified in positive mode: 4-hydroxynonenal, carbofuran, cerulenin, diethylphosphate, fenpropimorph, naphthalene and onchidal. The compound 4-hydroxynonenal was assigned to *m*/*z* 157.1225. This compound has been reported as a apoptotic agent in many cell cultures [48]. Carbofuran is a pesticide that is commonly used as an insecticide, acaricide and nematicide [49]. Cerulenin is an antifungal antibiotic that inhibits the growth of several species of mycobacteria [50]. Diethylphosphate is a known neurotoxin that is used as a pesticide [51]. Fenpropimorph is a fungicide [52]. Naphthalene has been shown to cause red blood cell haemolysis and may selectively injure the intrapulmonary airways’ nonciliated epithelial cells [53]. Onchidal is a naturally occurring neurotoxin [54].

## 3. Conclusions

The total dust concentration in the sewage treatment plant was very low, and no permissible dust concentration limits were exceeded at the workstations. Therefore, there is no need to use respiratory protection against particles.

The highest microbiological contamination of all tested places was discovered for Workstations 4 (sludge thickening building) and 5 (screenings storage). Still, the results reveal low (similar to the control atmospheric air) microbiological contamination at the tested workstations in the sewage treatment plant.

This is the first study to thoroughly analyse mycotoxins in the air and settled dust in a sewage treatment station. Three chemical compounds were detected in the air and sixteen in the settled dust from the referenced work environment. Compounds characteristic for lichen and plants and *Aspergillus*, *Penicillium* and *Fusarium* genera mould dominated the identified metabolites. Settled dust from the workstation in the sludge thickening building revealed high cytotoxicity to human lung epithelial cells A-549. It suggests the presence of non-biological inhalation hazards at the tested workstation—compounds from the group of antibiotics, pesticides and disinfectants, which may adversely affect employees’ health. Their presence might negatively affect biological sewage treatment and the natural environment and contribute to increasing antibiotic resistance of microorganisms in the future. 

## 4. Materials and Methods

### 4.1. Workstations in the Tested Sewage Treatment Plant

The tests were carried out in a treatment plant processing sewage from an area inhabited by ca. 820,000 people, designed for the maximum dry weather flow rate of 215,300 m^3^/d and 1,026,260 population equivalent load. It is a typical mechanical and biological sewage treatment plant with improved removal of biogenic compounds. The biological process is periodically supported with an iron coagulant and an external source of carbon. In 2020, over 163,000 m^3^ of sewage were supplied to the tested sewage treatment plant a day and nearly 7000 m^3^ of sewage per hour. The tests were carried out at six workstations described in Table 6.

### 4.2. Airborne Dust Concentration Measurement

A portable laser photometer DustTrak™ DRX Aerosol Monitor 8533 (TSI, Shoreview, MN, USA) was used to measure airborne dust concentration. Size-segregated mass concentrations of particulate matter (PM) corresponding to PM_1_ (particles and droplets with diameters < 1 μm), PM_2.5_ (<2.5 μm), PM_4_ (<4 μm), PM_10_ (<10 μm) and total PM (all particles from the measured diameter size range) were determined. The instrument’s detection range ranged from 0.001 to 150 mg m^−3^ for particles 0.1–15 μm in size. Zero calibration was performed prior to each experiment. The measurements were obtained at the height of 1.5 m from ground level in triplicates for each location, with a sampling rate of 3 L min^−1^ and a sampling interval of 5 s.

### 4.3. Microbial Contamination Analysis

Microorganism numbers were determined for the air and settled dust from the sewage treatment plant workstations.

Microbiological contamination of the air was determined using a MAS-100 Eco Air Sampler (Merck, Germany) according to the EN 13098 standard. Air samples of 20 L–100 L were collected onto MEA (Malt Extract Agar, Merck, Germany) medium with (0.1%) chloramphenicol (fungi); DG18 Agar (DG18 LAB -AGAR™, Biocorp, Poland) (xerophilic fungi); TSA (Triptic Soy Agar, Merck, Germany) with (0.2%) nystatin (bacteria); Columbia Blood Agar, (Oxoid, France) (haemolytic *Staphylococcus* spp.); Pochon’s agar (Labomix, Poland) with (0.2%) nystatin (actinomycetes); Chapman Agar (Merck, Germany) (mannitol-positive *Staphylococcus* spp.); King B medium (Hi Media Laboratories, India) (*Pseudomonas fluorescens*); and Violet Red Bile Glucose Agar (VRBG LAB-AGAR, Biocorp, Poland) (*Enterobacteriaceae*). Samples of air were collected from three locations at the height of 1.5 m during routine work (Table 6). Atmospheric air samples (external background) were collected at a distance of 0.5 km from the sewage treatment plant.

Samples of settled dust from working environments were microbiologically analysed. For this purpose, 10–15 g the dust samples from flat surfaces of technological devices were collected in sterile containers, mixed, and 0.1 g of each of the mixed samples was suspended in 9.9 mL of saline solution (0.85% NaCl). The samples were diluted from 10^−2^ to 10^−6^ in triplicates and plated onto the abovementioned media.

All samples (air, dust) were incubated at either 37 °C ± 2 °C for 24–48 h (*Enterobacteriaceae*, mannitol-positive *Staphylococcus* spp., haemolytic *Staphylococcus* spp.), 25 °C ± 2 °C for 5–7 days (fungi, xerophilic fungi, actinomycetes), or 30 °C ± 2 °C for 48 h (bacteria, *Pseudomonas fluorescens*).

After incubation, the colonies were counted, and the results were expressed in CFU m^−3^ for air and CFU g^−1^ for settled dust. The final result was calculated as the arithmetic mean of three independent repetitions.

### 4.4. Assessment of Microbial Diversity by High-Throughput Sequencing

Air and dust samples for DNA extraction were collected from Workstation 4. Air (7000 L) was passed through sterile gelatine filters (80 mm, 0.3 µL Sartorius, Goettingen, Germany) using AirPort MD 8 (Sartorius, Goettingen, Germany).

Genomic DNA was isolated using a modified method based on Genomic Mini AX Bacteria + set (A&A Biotechnology, Poland). Additional mechanical lysis of the samples in a FastPrep-24 device type was performed using zirconium oxide beads. After the isolation, the DNA was additionally treated using Anti-Inhibitor Kit (A&A Biotechnology, Poland). The presence of bacterial DNA in the tested samples was confirmed with the real-time PCR. The real-time PCR reaction was carried out in Mx3000P thermocycler (Stratagene, La Jolla, CA, USA), using SYBR Green dye as a fluorochrome. The isolated DNA concentration ranged from 2 to 30 µg mL^−1^. Universal starters [55] amplifying the 16s rRNA bacterial gene’s fragment were used in the reaction. Before the library of V3-V4 and ITS amplicons were prepared, the DNA eluates had been checked for their quality and quantity. The libraries were prepared according to the guidelines of 16S Metagenomic Sequencing Library Preparation Part # 15044223 Rev. B; Herculase II Fusion DNA Polymerase Nextera XT Index Kit V2 was used for the two-step PCR.

The libraries were prepared by Macrogen (South Korea). The library’s quality was checked according to Illumina qPCR Quantification Protocol Guide.

The sequencing was performed by Macrogen (South Korea) using the paired-end technology on Illumina MiSeq (2 × 300 bp) platform.

Bioinformatic analysis of the sequencing results was carried out for qualitative and quantitative taxonomic identification as described by [56]. The analysis was performed using CLC Genomic Workbench v. 12 (Qiagen) + Microbial Genomics Module Plugin v. 4.1 (Qiagen).

### 4.5. Analysis of Endotoxin in Dust Samples

3-hydroxy fatty acids (3-OH FAs), which are unique compounds within the conserved portion of lipopolysaccharides (LPS), were used to identify endotoxins in settled dust samples from the waste sorting plant. The samples were subjected to hydrolysis to obtain fatty acid methyl esters in a 2M methanolic HCl at 85 °C for 18 h. Then they were extracted to a solid phase. The material was derivatised to obtain silyl derivatives using bis(trimethylsilyl)-trifluoroacetamide (Merck) and pyridine at 60 °C for 20 min. A quantitative analysis was performed using deuterated 3-hydroxytetradecanoic acid.

The derivatives were analysed using gas chromatography-mass spectrometry (Agilent GC 7890B) at a DB-5ms column (Agilent) at 150–280 °C (6 °C/min), MS/MS in the SIM mode. The results were expressed in LPS nmol per mg of dust.

### 4.6. Secondary Metabolites

For secondary metabolite analyses, air samples (2000 L) from gelatine filters and 1 g of dust were dissolved in 8 and 10 mL extraction solvent (acetonitrile/water/acetic acid 79:20:1, *v*/*v*/*v*), respectively. Next, dust and air samples were extracted for 90 min and diluted in the same solvent volume prior to injection. Secondary metabolite concentrations were analysed and quantified using LC-MS/MS, as described by Sulyok et al. with further modification [57]. Parameters for liquid chromatography and mass spectrometry are described elsewhere [57]. The limits of detection (LOD) of extrolites are presented in supplementary materials Table S3.

### 4.7. Cell Culture and Cytotoxicity Testing

Human adenocarcinoma lung (alveolar) epithelial cell line A-549 (Cell Line Service GmbH, Germany) from passage 36 was used as a model cell line for dust and air pollution cytotoxicity testing [20]. In short, the cells were cultured (37 °C, 5% CO_2_) as a monolayer in Dulbecco’s modified Eagle’s medium:Ham’s F12 basic (1:1, *v*/*v*, CLS GmbH, Germany) with 2 mM glutamine, 5% foetal bovine serum (FBS; CLS GmbH, Germany) and 25 mM of HEPES (Sigma-Aldrich). After reaching 80% confluence, cells were detached, centrifuged (187× *g*, 5 min) and re-suspended in fresh culture medium. The number of cells and viability was assessed with a haemocytometer by trypan blue exclusion.

Dust samples were suspended in culture medium, mixed (stock dust concentration was 100 mg mL^−1^), extracted (40 min, 160 rpm), pH adjusted to neutral (7.0 ± 0.2) and double filtered (0.22 m, Membrane Solutions, Auburn, WA, USA). The final tested concentrations of extract were from 0.3 to 20 mg mL^−1^. Cytotoxicity was estimated with MTT (3-(4,5-dimethylthiazol-2-yl)-2,5-diphenyltetrazolium bromide) assay as described previously [58]. The cells were exposed to dust extract for 48 and 72 h (37 °C, 5% CO_2_). The morphology of A-549 cells was controlled under an inverted microscope Nikon Ts2 with contrast EMBOSS, integrated with a digital camera Jenoptic Subra Full HD Color. The IC50 was determined both from the plotted curves and according to the OECD Guidelines for the Testing of Chemicals [59,60].

### 4.8. UHPLC-Q-ToF-UHRMS Analysis

Dust samples (1 g) were suspended in 1 mL LC-MS-grade methanol purchased from Aldrich (Poland) and extracted (1 h, 150 rpm, ambient temperature). Methanol extracts were filtered through syringe filters (0.22 m) (Membrane Solutions, Auburn, WA USA), transferred into HPLC vials and inserted into Elute autosampler. A sample chamber of the autosampler was held at 5 °C for analysis time.

Instrumental configuration consisted of a Bruker Elute UHPLC system operated by Hystar 3.3 software and a mass spectrometer Bruker Impact II (Bruker Daltonik GmbH) ESI QTOF-MS equipped with Data Analysis 4.2 (Bruker Daltonik GmbH), TASQ (2021b) and Metaboscape (ver. 2021b). A Bruker UHPLC column Intensity Solo (C18 silica, 1.8 μm particles, 100 × 2.1 mm) with column guard was used for all analyses. Two mobile phases were: A = Water/methanol (99:1) with 5 mM ammonium formate and 0.01% formic acid, B = Methanol with 5 mM ammonium formate and 0.01% formic acid (*v*/*v*). All chemicals were of analytical reagent grade. Deionised water (18 MΩ.cm) was produced locally. Autosampler was held at a temperature of 4 °C. The volume of 5 μL of the extract was loaded on the column at a flow rate of 200 μL min^−1^, using 4% B. For Targetscreener bbCID measurements B percentage was changed with time: 1 min—18.3% B, 2.5 min—50%, 14–16 min 99.9%, 16.1–20 min—4%. The solvent flow was 200 μL min^−1^ from 0 to 1 min, gradually changing from 200 to 223 μL min^−1^ from 1 to 2.5 min and from 223 μL min^−1^ to 400 μL min^−1^ from 2.5 to 14 min, then from 400 μL min^−1^ to 480 μL min^−1^ from 14 to 19 min and back to 200 μL min^−1^ from 19.1 to 20 min. For AutoMSMS measurements, flows and percentages were identical as provided above with the exception that 99.9% B was held up to 25 min at 480 μL min^−1^ and 4% B was from 26.1 to 30 min. The column was held at 40 °C. The column exit was connected to ESI source. Internal calibration on 10 mM sodium formate (water: isopropanol 1:1 *v*/*v*) ions was performed automatically in Metaboscape using a syringe pump at an infusion flow rate of 0.12 mL h^−1^, using a high precision calibration (HPC) mode. Analyses in positive bbCID mode were carried out using the following parameters: 50–1000 *m*/*z*; capillary voltage: 4 kV; nebuliser: 4 bar; dry gas: 8 L min^−1^; drying gas temperature: 200 °C; hexapole voltage: 30 Vpp; funnel 1: 300 Vpp; funnel 2: 300 Vpp; pre-pulse storage time: 8 μs; transfer time: 60 μs. For autoMSMS mode *m*/*z* range was 50-1500, CID (collision-induced dissociation) was used with the following settings: absolute area threshold: 5000 counts; active exclusion 2 spectra; release after 0.3 min, isolation mass: for *m*/*z* = 100, the width was 4, for 300 it was 5, for 500 it was 6 and for 1000 it was 8): 15, 10, 5 eV; collision energy values was 30 eV. Targeted identification was performed in TASQ (ver. 2021b). The untargeted annotations were performed in Metaboscape (ver. 2021b) with a criterion of mass deviation (Δ*m*/*z*) under 2 ppm and mSigma value under 15 as the maximum acceptable deviation of the mass of the compound and the isotopic pattern, respectively. The molecular formulas were obtained using the Smart Formula tool and the C, H, N, O, P, S, Cl, Br, I and F elements. MSMS spectra were automatically matched against MSMS libraries: Bruker HMDB 2.0 library, MassBank of North America (MoNA) library [61] and NIST ver. 2014 MSMS library [62].

### 4.9. Statistical Analysis

Statistical analyses were conducted using Statistica 13.1 (Statsoft, Tulsa, OK, USA). Descriptive statistics for all variables of interest were calculated. Microorganism numbers in the air and dust samples as well as microclimatic conditions and dust concentrations were compared between the tested workstations using one-way analysis of variance (ANOVA) at a significance level of 0.05 following Levene’s test. When a statistical difference was detected (*p* < 0.05), means were compared using Tukey’s post-hoc procedure at a 0.05 significance level.

For cytotoxicity testing, two-way analysis of variance (ANOVA) was conducted using OriginPro 6.1 (Northampton, MA, USA) software to evaluate the experimental data. The significant differences between the means were compared using Scheffe’s multiple comparison test, and they were accepted to be significant at *p* < 0.05.

Linear regression analysis was used to determine the correlation between the number of microorganisms in the air and settled dust at particular locations within the cattle farm. The significance tests were performed at the 0.05 significance level using a literature correlation scale [63].

## Figures and Tables

**Table 1:** Microclimate parameters in the sewage treatment plant.

No.*	Temperature (°C)	Relative Humidity (%)	Airflow Rate (m s^−1^)
1	M:17.6 ^b^	M:71.0 ^a^	M:0.87 ^d^
	SD:0.3	SD:1.8	SD:0.28
2	M:17.4 ^b^	M:73.1 ^a^	M:0.55 ^cd^
	SD:0.3	SD:1.5	SD:0.34
3	M:20.1 ^a^	M:65.3 ^bd^	M:0.39 ^bc^
	SD:1.0	SD:3.2	SD:0.11
4	M:21.5 ^a^	M:57.7 ^c^	M:0.03 ^a^
	SD:0.5	SD:1.3	SD:0.01
5	M:24.8 ^cd^	M:71.1 ^ab^	M:0.31 ^abc^
	SD:0.7	SD:4.7	SD:0.21
6	M:26.4 ^d^	M:69.8 ^ab^	M:0.37 ^abc^
	SD:2.7	SD:5.1	SD:0.10
EB	M:22.1 ^ac^	M:58.2 ^cd^	M:0.03 ^ab^
	SD:0.2	SD:1.0	SD:0.02

No.*—tested workstation number; EB—external background (atmospheric air sampled 0.25 km from the sorting plant); M—mean; SD—standard deviation; a, b, c, d—significant statistical differences occur for the means marked with different letters within the particular parameter (column), ANOVA, *p* < 0.05; Tukey’s HSD test, *p* < 0.05.

**Table 2:** Airborne dust concentration in the sewage treatment plant.

No.	Dust Concentration Corresponding to Different Fractions, mg m^−3^				
	PM_1_	PM_2.5_	PM_4_	PM_10_	PM_total_
1	M:0.040 ^a^	M:0.040 ^a^	M:0.041 ^b^	M:0.042 ^c^	M:0.044 ^a^
	SD:0.081	SD:0.081	SD:0.082	SD:0.087	SD:0.099
2	M:0.037 ^ac^	M:0.038 ^ab^	M:0.038 ^ab^	M:0.040 ^abc^	M:0.041 ^ab^
	SD:0.004	SD:0.004	SD:0.005	SD:0.007	SD:0.008
3	M:0.036 ^ac^	M:0.037 ^ac^	M:0.037 ^ab^	M:0.038 ^ab^	M:0.040 ^ab^
	SD:0.003	SD:0.003	SD:0.003	SD:0.005	SD:0.011
4	M:0.032 ^b^	M:0.033 ^b^	M:0.035 ^ac^	M:0.041 ^abc^	M:0.043 ^a^
	SD:0.006	SD:0.006	SD:0.006	SD:0.009	SD:0.013
5	M:0.044 ^b^	M:0.044 ^b^	M:0.045 ^c^	M:0.047 ^d^	M:0.047 ^b^
	SD:0.005	SD:0.005	SD:0.005	SD:0.006	SD:0.007
6	M:0.033 ^bc^	M:0.034 ^bc^	M:0.034 ^ac^	M:0.037 ^ad^	M:0.039 ^ab^
	SD:0.006	SD:0.006	SD:0.006	SD:0.008	SD:0.012
EB	M:0.030 ^a^	M:0.030 ^a^	M:0.031 ^b^	M:0.034 ^bc^	M:0.036 ^a^
	SD:0.006	SD:0.006	SD:0.007	SD:0.010	SD:0.017

No.—tested workstation number; EB—external background (atmospheric air sampled 0.25 km from the sorting plant); M—mean; SD—standard deviation; a, b, c, d—statistically significant differences occur for the means marked with different letters within the particular dust fraction (ANOVA, α = 0.05; Tukey’s HSD test, α = 0.05).

**Table 3:** Microorganisms count in the sewage treatment plant air.

No.	Microorganisms Count, CFU m^−3^							
	Bacteria	Actinomycetes	Mannitol-Positive Staphylococci	*Enterobacteriaceae*	*Pseudomonas fluorescens*	Haemolytic Staphylococci	Fungi	Xerophilic Fungi
1	M:7.75 × 10^2 A^	M:0.00 ^A^	M:2.50 × 10^0 A^	M:2.50 × 10^0 A^	M:4.00 × 10^1 B^	M:5.75 × 10^1 A^	M:2.75 × 10^3 A^	M:1.89 × 10^3 A^
	SD:5.74 × 10^1^	SD: 0.00	SD:5.00 × 10^0^	SD:5.00 × 10^0^	SD:8.16 × 10^0^	SD:5.00 × 10^0^	SD:5.11 × 10^2^	SD:7.54 × 10^2^
2	M:3.75 × 10^2 A^	M:1.50 × 10^1 B^	M:2.50 × 10^0 A^	M:1.50 × 10^1 A^	M:1.50 × 10^1 A^	M:5.75 × 10^1 A^	M:2.69 × 10^3 A^	M:1.87 × 10^3 A^
	SD:1.49 × 10^2^	SD:1.00 × 10^1^	SD:5.00 × 10^0^	SD:1.00 × 10^1^	SD:1.00 × 10^1^	SD:1.71 × 10^1^	SD:5.36 × 10^2^	SD:3.74 × 10^2^
3	M:3.05 × 10^2 A^	M:0.00 ^A^	M:7.50 × 10^0 A^	M:1.50 × 10^1 A^	M:7.50 × 10^0 A^	M:5.00 × 10^1 A^	M:2.13 × 10^3 A^	M:1.13 × 10^3 A^
	SD:6.40 × 10^1^	SD:0.00	SD:9.57 × 10^0^	SD:1.00 × 10^1^	SD:9.57 × 10^0^	SD:1.41 × 10^1^	SD:4.46 × 10^2^	SD:1.13 × 10^2^
4	M:3.71 × 10^3 B^	M:0.00 ^A^	M:6.50 × 10^1 B^	M:1.50 × 10^1 A^	M:2.00 × 10^1 AB^	M:2.10 × 10^2 A^	M:1.91 × 10^3 A^	M:1.55 × 10^3 A^
	SD:7.57 × 10^2^	SD:0.00	SD:3.00 × 10^1^	SD:1.00 × 10^1^	SD:1.63 × 10^1^	SD:1.01 × 10^2^	SD:4.38 × 10^2^	SD:1.54 × 10^2^
5	M:6.28 × 10^2 A^	M:0.00 ^A^	M:1.00 × 10^1 A^	M:1.98 × 10^2 C^	M:1.00 × 10^1 A^	M:3.25 × 10^2 A^	M:3.23 × 10^4 B^	M:1.26 × 10^4 AB^
	SD:1.61 × 10^2^	SD:0.00	SD:1.15 × 10^1^	SD:1.07 × 10^3^	SD:1.15 × 10^1^	SD:4.50 × 10^2^	SD:2.34 × 10^4^	SD:1.84 × 10^4^
6	M:3.38 × 10^2 A^	M:0.00 ^A^	M:6.75 × 10^1 B^	M:6.85 × 10^1 B^	M:1.00 × 10^1 A^	M:5.00 × 10^1 A^	M:2.80 × 10^4 AB^	M:2.39 × 10^4 B^
	SD:5.06 × 10^1^	SD:0.00	SD:2.22 × 10^1^	SD:9.50 × 10^1^	SD:1.15 × 10^1^	SD:8.16 × 10^0^	SD:1.73 × 10^4^	SD:1.97 × 10^4^
EB	M:2.15 × 10^2 A^	M:1.50 × 10^1 B^	M:2.50 × 10^0 A^	M:1.00 × 10^1 A^	M:0.00 ^A^	M:1.50 × 10^1 A^	M:1.02 × 10^4 AB^	M:1.01 × 10^4 AB^
	SD:6.81 × 10^1^	SD:1.00 × 10^1^	SD:5.00 × 10^0^	SD:0.00 × 10^0^	SD:0.00	SD:1.00 × 10^1^	SD:1.08 × 10^4^	SD:1.09 × 10^4^

No.—tested workstation number; EB—external background (atmospheric air sampled 0.25 km from the sorting plant); M—mean; SD—standard deviation; A, B, C—the means marked with the same letter in the same column do not differ in a statistically significant way (Tukey’s HSD test; *p* < 0.05).

**Table 4:** The concentration of metabolites detected in the air and dust settled at the workstations in the sewage treatment plant.

Metabolite	Concentration	
	Air, ng m^−3^	Dust, ng g^−1^
3-Nitropropionic acid	1.98	5.87
Quinolactacin A	<LOD	1.58
Citreohybridinol	<LOD	3.49
Flavoglaucin	1.54	1.65
Pentoxifylline	<LOD	0.90
Beauvericin	<LOD	0.24
Enniatin A1	<LOD	0.58
Enniatin B	<LOD	2.32
Enniatin B1	<LOD	1.21
Lecanoric acid	<LOD	129
Usnic acid	<LOD	14.0
Prunasin	<LOD	23.1
Asperglaucide	<LOD	2.30
Asperphenamate	0.46	7.37
cyclo(L-Pro-L-Tyr)	<LOD	18.1
Emodin	<LOD	2.59

<LOD—below the limit of detection.

**Table 5:** Identification parameters of toxic compounds found in dust.

Compound	RT, min	Measured *m/z*	Mass Error, ppm	Molecular Formula	Ion Formula	Meas. Mode	mSigma	MS/MS
Didecyldimethylammonium chloride	12.11	326.38	0.49	C_22_H_48_ClN	[M−Cl]^+^	+bbCID	8.0	yes
4-Hydroxynonenal	6.15	157.12	1.25	C_9_H_16_O_2_	[M+H]^+^	+AutoMSMS	5.6	yes
Carbofuran	29.92	222.11	0.94	C_12_H_15_NO_3_	[M+H]^+^	+AutoMSMS	12.1	yes
Cerulenin	0.08	224.13	0.96	C_12_H_17_NO_3_	[M+H]^+^	+AutoMSMS	12.8	yes
Diethylphosphate	5.78	155.05	0.91	C_4_H_11_O_4_P	[M+H]^+^	+AutoMSMS	6.1	yes
Fenpropimorph	13.42	304.26	0.64	C_20_H_33_NO	[M+H]^+^	+AutoMSMS	0.8	yes
Naphthalene	6.73	129.07	0.98	C_10_H_8_	[M+H]^+^	+AutoMSMS	6.3	yes
Onchidal	15.62	277.18	0.63	C_17_H_24_O3	[M+H]^+^	+AutoMSMS	-	no

**Table 6:** Characteristics of the workstations tested in the sewage treatment plant.

No.	Workstation Name	Description
1	Primary settlement tank inlet	Rectangular primary settlement tank (6 tanks ca. 4000 m^3^ volume each) featured with scrapers. The final stage of sewage mechanical treatment is carried out here. The sludge separated on the bottom is scraped to the hoppers from where it is removed to the fermentation chambers.
2	Activated sludge chamber inlet	Activated sludge rectangular chambers (7 chambers ca. 19,900 m^3^ volume each). Biological treatment of the sewage is carried out here. Organic and biogenic compounds (nitrogen, phosphorus) from the sewage are decomposed by the microorganisms in the activated sludge. The process varies, depending on a number of factors, including oxygen content, temperature, bacteria genus, supplied sewage characteristics and the adopted treatment method.
3	Aeration chambers	Oxygen (nitrification) zone composed of two piston flow chambers, equipped with a fine bubble aeration system.
4	Sludge thickening building	The sludge (primary and surplus) is thickened before being fed for further treatment. The primary sludge is gravitationally thickened in primary sludge hoppers and optionally in gravitational hoppers (3 hoppers 539 m^3^ volume each). The surplus sludge is thickened at sludge belt thickeners (5 thickeners 91.5 m^3^/h capacity each) using polyelectrolyte. The thickened sludge is stabilised through methane fermentation.
5	Screenings storage	The screenings obtained through mechanical treatment are deposited in a separated storage area. A coarse grate with 100 mm mesh size is used in the sewage treatment plant—it protects fine grates against large items supplied by combined sewers. Then the sewage is divided into 1 ÷ 4 lines in the main inlet chamber. Each line is handled by a set of two grates. Hook and slot grates work at two lines (6 mm clearance) and disc screen sets on the other two lines (55 mm clearance), with mills and lamellar grating.
6	Sludge lagoons	The wastewater from the grate room flows into four non-aerated sand traps. The sand collected at the bottom is scraped to the hoppers and pumped as a pulp into scrapers and then to the chamber scrubbers. The removed sand as a mineral (containing less than 3% of organic compounds) is deposited in sludge lagoons.

Microclimatic conditions (temperature, relative humidity, air velocity) were measured using a thermo-anemometer VelociCalc^®^ Multi-Function Velocity Meter 9545 (TSI, Shoreview, MN, USA).

## Data Availability

The data presented in this study are available on request from the corresponding author.

## Supplementary Materials

The following are available online at https://www.mdpi.com/article/10.3390/toxins13100691/s1. Table S1: Diversity of bacterial/archaeal genera in dust and air samples. Table S2: Diversity of fungal genera in dust and air samples. Table S3: The limits of detection of extrolites in dust samples. Figure S1: EICs of DDAC-C10 ions measured with bbCID Targetscreener positive method.
