# Supplementary material for: Microbiological and Toxicological Hazards in Sewage Treatment Plant Bioaerosol and Dust
Source: Toxins (Basel). 2021 Sep 28;13(10):691. doi: 10.3390/toxins13100691 (PMC8540054; doi:10.3390/toxins13100691)
Supplement: Supplementary file 1 [file toxins-13-00691-s001.zip › toxins-1375486-supplementary.pdf]

Supplementary material: Microbiological and Toxicological Hazards in Sewage Treatment Plant Bioaerosol and Dust

Justyna Szulc, Małgorzata Okrasa, Katarzyna Majchrzycka, Michael Sulyok, Adriana Nowak, Tomasz Ruman, Joanna Nizioł, Bogumiła Szponar and Beata Gutarowska

Table S1. Diversity of bacterial/archeal genera in dust and air samples.

Domain	Phylum	Genus	Abundance of Bacterial/Archeal Genera in Samples, %*	
			Air	Dust
Archaea	Euryarchaeota	*Methanosaeta*	0.002	0.002
Bacteria	Thermi	*Deinococcus*	0.023	3.000
	Acidobacteria	Unidentified	0.048	0.186
	Actinobacteria	*Actinomyces*	0.005	0.000
		Agrococcus	0.000	0.005
		Agromyces	0.000	0.005
		Amycolatopsis	0.000	0.027
		Arcanobacterium	0.000	0.010
		Arthrobacter	0.005	0.094
		Bifidobacterium	6.000	5.000
		Brevibacterium	0.002	0.360
		Candidatus Microthrix	0.345	0.941
		Cellulomonas	0.041	0.000
		Cellulosimicrobium	59.000	0.706
		Collinsella	0.007	0.031
		Corynebacterium	0.025	1.000
		Gordonia	0.301	3.000
		Kocuria	0.002	0.046
		Kribbella	0.000	0.007
		Mycobacterium	0.411	0.832
		Nesterenkonia	0.007	0.174
		Nocardia	0.000	0.015
		Patulibacter	0.000	0.140
		Piscicoccus	0.000	0.106
		Propionibacterium	0.032	0.051
		Propionicimonas	0.005	0.029
		Pseudonocardia	0.000	0.031
		Rhodococcus	0.002	0.699
		Saccharomonospora	0.000	0.002
		Solirubrobacter	0.005	0.000
		Terracoccus	0.096	1.000
		Thermobispora	0.000	0.010
		Thermocrispum	0.000	0.007
		Tsukamurella	0.007	0.106
		Verrucosispora	0.000	0.005
		Unidentified	1.615	5.864
	Armatimonadetes	Unidentified	0.005	0.042
	Bacteroidetes	*Bacteroides*	0.009	0.007

	Chryseobacterium	0.023	10.000
	Dysgonomonas	0.005	0.000
	Flavisolibacter	0.009	0.005
	Flavobacterium	0.014	0.000
	Hymenobacter	0.005	0.015
	Niabella	0.011	0.005
	Paludibacter	0.009	0.010
	Parabacteroides	0.000	0.005
	Pedobacter	0.000	0.046
	Pontibacter	0.000	0.015
	Rubricoccus	0.000	0.015
	Sediminibacterium	0.005	0.005
	Wautersiella	0.000	0.002
	Weeksella	0.000	0.058
	Unidentified	0.661	0.561
Chlamydiae	*Candidatus Rhabdochlamydia*	0.005	0.000
	Unidentified	0.014	0.000
Chlorobi	Unidentified	0.117	0.097
Chloroflexi	Unidentified	0.168	0.539
Cyanobacteria	*Microcoleus*	0.000	0.015
	Nostoc	0.000	0.022
	Unidentified	0.070	0.907
Firmicutes	*Aerococcus*	0.000	0.019
	Anaerosinus	0.005	0.000
	Anoxybacillus	0.000	0.005
	Bacillus	0.075	0.440
	Blautia	0.011	0.002
	Brevibacillus	0.000	0.010
	Caldicoprobacter	0.000	0.005
	Caloramator	0.000	0.034
	Clostridium	0.041	0.331
	Dorea	0.005	0.005
	Enterococcus	0.009	0.181
	Ethanoligenens	0.000	0.007
	Exiguobacterium	0.005	0.293
	Facklamia	0.002	0.022
	Faecalibacterium	0.000	0.010
	Finegoldia	0.005	0.000
	Geobacillus	0.000	0.019
	Granulicatella	0.005	0.000
	Jeotgalicoccus	0.005	0.075
	Lachnospira	0.000	0.010
	Lactobacillus	0.021	0.019
	Lactococcus	0.005	0.010
	Lutispora	0.000	0.012
	Lysinibacillus	0.000	0.031
	Macrococcus	0.002	0.160
	Megamonas	0.009	0.000
	Natronobacillus	0.000	0.010
	Oscillospira	0.009	0.000
	Paenibacillus	0.000	0.005

	Pediococcus	0.005	0.002
	Planifilum	0.000	0.017
	Planococcus	0.005	0.534
	Roseburia	0.000	0.017
	Ruminococcus	0.023	0.077
	Rummeliibacillus	0.002	0.099
	Salinicoccus	0.005	0.138
	Sporomusa	0.000	0.005
	Staphylococcus	0.014	0.626
	Streptococcus	0.053	0.039
	Syntrophomonas	0.053	0.060
	Tepidibacter	0.000	0.044
	Tepidimicrobium	0.000	0.022
	Trichococcus	0.000	0.053
	Turicibacter	0.048	0.909
	Vagococcus	0.000	0.005
	Unidentified	1.524	17.413
Fusobacteria	*Leptotrichia*	0.000	0.007
	Unidentified	0.010	0.005
Gemmatimonadetes	Unidentified	0.000	0.010
Nitrospirae	*Nitrospira*	0.014	0.048
Planctomycetes	Unidentified	0.024	0.063
Proteobacteria	*Achromobacter*	0.053	0.000
	Acinetobacter	0.050	4.000
	Agrobacterium	0.007	0.184
	Allochromatium	0.171	0.075
	Aquabacterium	0.132	0.005
	Aquicella	0.002	0.007
	Arcobacter	0.119	0.000
	Aurantimonas	0.002	0.007
	Balneimonas	0.005	0.044
	Bdellovibrio	0.005	0.015
	Blastomonas	0.023	0.022
	Brachymonas	0.005	0.000
	Brevundimonas	0.011	0.363
	Burkholderia	0.892	0.029
	Dechloromonas	0.062	0.015
	Delftia	0.000	0.007
	Desulfobulbus	0.000	0.005
	Devosia	0.005	0.068
	Dokdonella	0.050	0.000
	Eikenella	0.005	0.000
	Enhydrobacter	0.014	0.708
	Erwinia	0.000	0.000
	Erythrobacter	0.000	0.005
	Escherichia	0.032	0.060
	Gyrocarpus	0.000	0.005
	Hyphomicrobium	0.014	0.077
	Janthinobacterium	0.000	0.005
	Kaistobacter	0.005	0.058
	Kingella	0.406	0.118

		Lardizabala	0.000	0.007
		Luteimonas	0.000	0.024
		Methylobacterium	0.000	0.012
		Mycoplana	0.002	0.247
		Myristica	0.000	0.002
		Nannocystis	0.011	0.000
		Nitrosomonas	0.005	0.000
		Ochrobactrum	0.018	0.109
		Pantoea	0.069	0.551
		Perlucidibaca	0.116	0.007
		Pirellula	0.000	0.012
		Planctomyces	0.005	0.012
		Plesiomonas	0.000	0.063
		Propionivibrio	0.005	0.000
		Proteus	0.000	0.034
		Providencia	0.000	0.007
		Pseudochrobactrum	0.007	0.053
		Pseudomonas	12.000	4.000
		Psychrobacter	0.016	4.000
		Rhodobacter	0.000	0.005
		Rhodoferax	0.073	0.015
		Rhodoplanes	0.000	0.027
		Roseococcus	0.000	0.005
		Rubellimicrobium	0.002	0.022
		Serratia	0.009	2.000
		Skermanella	0.005	0.070
		Sphingobium	0.000	0.017
		Sphingomonas	0.016	4.000
		Stenotrophomonas	8.000	1.039
		Syntrophus	0.100	0.007
		Thermomonas	0.000	0.024
		Tolumonas	0.005	0.000
		Wohlfahrtiimonas	0.000	0.005
		Unidentified	4.619	14.188
	Spirochaetes	*Treponema*	0.073	0.181
		Unidentified	0.064	0.015
	Synergistetes	*Aminiphilus*	0.011	0.002
		Unidentified	0.062	0.075
	Tenericutes	Unidentified	0.005	0.000
	Thermotogae	*Fervidobacterium*	0.018	0.048
		Kosmotoga	0.000	0.015
		Unidentified	0.010	0.019
	Verrucomicrobia	*Chthoniobacter*	0.000	0.005
		Luteolibacter	0.009	0.012
		Opitutus	0.005	0.000
		Prosthecobacter	0.018	0.000
		Verrucomicrobium	0.009	0.000
		Unidentified	0.016	0.024
	Unidentified		0.865	3.028
	Unassignated		0.064	0.169

*percentage share of studied genera greater than 0.001%.

Table S2. Diversity of fungal genera in dust and air samples.

Phylum	Order	Genera	Abundance of Fungal Genera in Samples. %*	
			Air	Dust
Ascomycota	*Lichenostigmatales*	*Phaeococcomyces*	0.000	0.014
	Ascomycota Incertae sedis	*Anguillospora*	0.008	0.005
		Engyodontium	0.003	0.001
		Radulidium	0.007	0.012
	Botryosphaeriales	*Diplodia*	0.000	0.008
		unidentified	0.001	0.015
	Capnodiales	*Arthrocatena*	0.000	0.005
		Camarosporula	0.005	0.000
		Capnobotryella	0.003	0.000
		Catenulostroma	0.012	0.000
		Cladosporium	14.000	6.000
		Devriesia	0.025	0.001
		Dissoconium	1.000	0.001
		Dothistroma	0.001	0.001
		Extremus	0.013	0.001
		Mycosphaerella	7.000	12.000
		Neocatenulostroma	0.004	0.033
		Neodevriesia	0.017	0.000
		Periconiella	0.003	0.000
		Phaeothecoidea	0.000	0.012
		Rachicladosporium	0.022	0.000
		Ramichloridium	0.003	0.000
		Ramularia	0.114	0.184
		Sphaerulina	0.121	0.013
		Toxicocladosporium	0.016	0.001
		Uwebraunia	0.177	0.000
		Vermiconia	0.017	0.000
		Verrucocladosporium	0.000	0.007
		Zygophiala	0.049	0.000
		Zymoseptoria	0.023	0.007
		unidentified	0.070	0.094
	Dothideales	*Aureobasidium*	0.099	0.162
		Endoconidioma	0.052	0.000
		Hormonema	0.000	0.016
		unidentified	0.000	0.015
	Dothideomycetes	*Arthrographis*	0.000	0.077
		Leptospora	0.114	0.115
		Perusta	0.000	0.007
		Thyrostroma	0.000	0.003
	Myriangiales	*Endosporium*	0.017	0.000
		unidentified	0.000	0.008
	Pleosporales	*Acicuseptoria*	0.000	0.012
		Alternaria	5.000	4.000
		Angustimassarina	0.000	0.014
		Biappendiculispora	0.000	0.006
		Bipolaris	0.074	0.005

	Camarosporium	0.000	0.001
	Chalastospora	0.787	3.000
	Corynespora	0.000	0.003
	Dendrothyrium	0.000	0.002
	Dendryphiella	0.017	0.000
	Dendryphion	0.005	0.035
	Didymella	2.000	4.000
	Keissleriella	0.000	0.009
	Leptosphaeria	0.000	0.002
	Lophiostoma	0.003	0.013
	Mycopappus	0.000	0.005
	Neoascochyta	0.341	5.000
	Neosetophoma	0.003	0.029
	Paraconiothyrium	0.000	0.003
	Paradendryphiella	0.000	0.008
	Paraphaeosphaeria	0.000	0.002
	Periconia	0.587	0.127
	Phaeosphaeria	0.003	0.006
	Plenodomus	0.000	0.002
	Pleurophoma	0.000	0.005
	Preussia	0.000	0.008
	Prosthemium	0.000	0.005
	Pyrenochaeta	0.000	0.002
	Pyrenochaetopsis	0.009	0.002
	Pyrenophora	0.000	0.267
	Sclerostagonospora	0.012	0.080
	Setomelanomma	0.000	0.014
	Setoseptoria	0.005	0.002
	Spegazzinia	0.008	0.003
	Sphaerellopsis	0.000	0.002
	Stagonospora	0.000	0.006
	Stemphylium	0.059	0.353
	Torula	0.095	0.042
	Westerdykella	0.000	0.007
	unidentified	3.854	1.132
	Venturia	0.053	0.007
	unidentified	0.049	0.050
Chaetothyriales	*Cladophialophora*	0.000	0.006
	Cyphellophora	0.000	0.035
	Exophiala	0.061	0.409
	Knufia	0.034	0.009
	Rhinocladiella	0.000	0.003
	unidentified	0.039	0.045
Eurotiales	*Aspergillus*	2.000	2.000
	Byssochlamys	0.003	0.046
	Hamigera	0.000	0.002
	Penicillium	3.000	3.000
	Talaromyces	0.059	0.036
	Thermomyces	0.026	0.080
	unidentified	0.026	0.012
Onygenales	*Aphanoascus*	0.003	0.000

		Ascosphaera	0.000	0.008
		Chrysosporium	0.008	0.075
		Eremascus	0.000	0.007
		Leucothecium	0.000	0.001
		unidentified	0.001	0.003
	Lecanorales	*Lecania*	0.031	0.032
		Scoliciosporum	0.000	0.301
		unidentified	0.000	0.002
	Pertusariales	unidentified	0.000	0.005
	Teloschistales	*Athallia*	0.005	0.000
		unidentified	0.000	0.003
	Erysiphales	*Blumeria*	0.942	0.138
		Erysiphe	0.013	0.089
		Golovinomyces	0.051	0.000
		Neoerysiphe	0.000	0.002
		Podosphaera	0.101	0.003
		Sawadaea	0.012	0.000
	Helotiales	*Articulospora*	0.009	0.027
		Botrytis	0.698	2.000
		Calloria	0.003	0.013
		Calycina	0.005	0.000
		Chalara	0.005	0.002
		Cistella	0.013	0.002
		Claussenomyces	0.022	0.003
		Cudoniella	0.000	0.002
		Encoelia	0.137	0.007
		Glarea	0.000	0.030
		Hymenoscyphus	0.012	0.005
		Hyphodiscus	0.003	0.000
		Lachnum	0.000	0.015
		Lanzia	0.000	0.008
		Moellerodiscus	0.000	0.007
		Oidiodendron	0.009	0.000
		Phialocephala	0.000	0.005
		Pyrenopeziza	0.000	0.003
		Sclerotinia	0.000	0.006
		Streptobotrys	0.000	0.005
		Tetrachaetum	0.000	0.035
		Torrendiella	0.000	0.002
		Valdensinia	0.018	0.005
		Vibrissea	0.000	0.002
		Xenopolyscytalum	0.009	0.000
		unidentified	0.096	0.118
	Leotiomycetes	*Pseudeurotium*	0.000	0.007
		Pseudogymnoascus	0.000	0.006
		unidentified	0.008	0.005
	Phacidiales	*Phacidium*	0.000	0.001
	Rhytismatales	unidentified	0.000	0.005
	Thelebolales	*Thelebolus*	0.000	0.039
	unidentified	unidentified	0.000	0.003
	Orbiliales	*Dactylella*	0.005	0.000

	Orbilia	0.013	0.002
Pezizales	*Cephaliophora*	0.000	0.204
	Desmazierella	0.023	0.010
	Heydenia	0.000	0.007
	Peziza	0.014	0.000
	Pseudoplectania	0.005	0.000
	Tricharina	0.004	0.000
	unidentified	0.033	0.006
Pezizomycotina	*Hymenula*	0.000	0.002
Saccharomycetales	*Blastobotrys*	0.000	0.053
	Candida	2.000	0.206
	Citeromyces	0.000	2.000
	Cyberlindnera	0.026	0.008
	Debaryomyces	0.003	0.031
	Diutina	0.000	0.014
	Hanseniaspora	0.003	0.108
	Kazachstania	0.009	0.001
	Kuraishia	0.000	0.002
	Pichia	0.005	0.052
	Saccharomyces	0.013	0.003
	Torulaspora	0.000	0.001
	Wickerhamomyces	0.003	0.006
	unidentified	0.001	0.041
Schizosaccharomycetales	*Schizosaccharomyces*	0.000	0.003
Boliniales	unidentified	0.001	0.008
Coniochaetales	*Coniochaeta*	0.001	0.005
	Lecythophora	0.000	0.001
Diaporthales	*Plagiostoma*	0.000	0.007
	unidentified	0.001	0.0014
Glomerellales	*Acrostalagmus*	0.003	0.000
	Gibellulopsis	0.000	0.009
	Lectera	0.023	0.008
	Musicillium	0.003	0.000
	Plectosphaerella	0.004	0.046
	unidentified	0.001	0.003
Hypocreales	*Acremonium*	0.177	0.124
	Beauveria	0.000	0.096
	Clonostachys	0.003	0.001
	Cordyceps	0.118	0.105
	Cylindrium	0.007	0.000
	Fusarium	0.000	0.030
	Gibberella	0.112	0.038
	Isaria	0.068	0.350
	Lecanicillium	0.000	0.344
	Metarhizium	0.004	0.008
	Microcera	0.000	0.003
	Nectria	0.003	0.003
	Neotyphodium	0.000	0.002
	Paecilomyces	0.001	0.015
	Pleonectria	0.001	0.006
	Protocrea	0.010	0.000

		Purpureocillium	0.012	0.131
		Sarocladium	0.144	0.165
		Simplicillium	0.000	0.032
		Stachybotrys	0.016	0.002
		Stilbella	0.000	0.003
		Stylonectria	0.005	0.000
		Tolypocladium	0.005	0.000
		Trichoderma	0.135	0.036
		unidentified	0.652	0.984
	Microascales	*Ceratocystis*	0.000	0.023
		Gamsia	0.000	0.059
		Microascus	0.079	0.408
		Parascedosporium	0.000	0.120
		Petriella	0.000	0.003
		Scedosporium	0.004	0.035
		Scopulariopsis	0.000	0.002
		unidentified	0.024	0.043
	Myrmecridiales	*Myrmecridium*	0.070	0.027
	Pleurotheciales	*Phaeoisaria*	0.000	0.020
	Sordariales	*Chaetomium*	0.186	0.238
		Humicola	0.000	0.040
		Lasiosphaeria	0.004	0.000
		Myceliophthora	0.017	0.016
		Neurospora	0.000	0.020
		Phialemonium	0.000	0.028
		Podospora	0.000	0.008
		Sordaria	0.000	0.002
		unidentified	0.016	0.003
	Sordariomycetes	*Rhodoveronaea*	0.000	0.002
	Togniniales	*Phaeoacremonium*	0.294	0.023
	Trichosphaeriales	*Nigrospora*	0.157	0.028
	Xylariales	*Annulohypoxylon*	0.005	0.002
		Arthrinium	0.311	0.132
		Castanediella	0.003	0.000
		Cryptosphaeria	0.013	0.015
		Diatrypella	0.000	0.002
		Eutypa	0.000	0.002
		Hansfordia	0.007	0.006
		Hypoxylon	0.085	0.003
		Microdochium	0.003	0.006
		Monographella	0.111	0.963
		Nodulisporium	0.000	0.002
		Polyscytalum	0.003	0.000
		Truncatella	0.012	0.022
		unidentified	0.005	0.009
	Taphrinales	*Protomyces*	0.001	0.008
		Taphrina	0.044	0.457
Basidiomycota	*Agaricales*	*Agaricus*	0.217	0.022
		Agrocybe	0.018	0.061
		Amanita	0.264	0.002
		Baeospora	0.027	0.000

	Bovista	0.066	0.006
	Calvatia	0.000	0.002
	Chlorophyllum	0.088	0.021
	Clitocybe	0.005	0.003
	Clitopilus	0.026	0.015
	Collybia	0.094	0.018
	Conocybe	0.030	0.025
	Coprinellus	1.000	0.646
	Coprinopsis	0.553	0.324
	Coprinus	0.281	0.014
	Cortinarius	0.055	0.000
	Cristinia	0.003	0.000
	Cryptomarasmius	0.000	0.006
	Cuphophyllus	0.003	0.000
	Echinoderma	0.031	0.003
	Entoloma	0.003	0.000
	Flammulina	0.004	0.000
	Galerina	0.029	0.003
	Gerhardtia	0.003	0.000
	Gymnopus	0.207	0.250
	Hebeloma	0.003	0.000
	Hohenbuehelia	0.003	0.000
	Hygrophorus	0.005	0.000
	Hypholoma	3.000	0.271
	Inocybe	0.013	0.005
	Kuehneromyces	0.723	0.015
	Lacrymaria	0.023	0.006
	Lepiota	0.043	0.015
	Lepista	0.078	0.068
	Leucoagaricus	0.005	0.007
	Lycoperdon	0.566	0.060
	Lyophyllum	0.101	0.000
	Macrolepiota	0.001	0.003
	Marasmius	0.042	0.070
	Melanoleuca	0.013	0.000
	Merismodes	0.000	0.002
	Mucronella	0.007	0.000
	Mycena	0.412	0.018
	Mycetinis	0.007	0.005
	Ossicaulis	0.009	0.000
	Panaeolus	0.000	0.008
	Panellus	0.004	0.000
	Paralepista	0.039	0.000
	Pholiota	0.213	0.040
	Pleurotus	0.515	0.006
	Pluteus	0.077	0.020
	Psathyrella	0.640	0.332
	Psilocybe	0.001	0.558
	Rhodocollybia	0.087	0.012
	Rhodocybe	0.039	0.003
	Rugosomyces	0.014	0.003

		Strobilurus	0.027	0.003
		Stropharia	0.062	0.031
		Tephrocybe	0.007	0.000
		Tricholoma	0.092	0.009
		Tricholomopsis	0.021	0.000
		Tubaria	0.003	0.000
		Tulostoma	0.012	0.003
		Typhula	0.016	0.000
		Volvariella	0.003	0.000
		Volvopluteus	0.014	0.002
		Xeromphalina	0.008	0.000
		unidentified	0.664	0.932
	Agaricomycetes	*Oxyporus*	0.033	0.024
	Amylocorticiales	*Amylocorticiellum*	0.014	0.000
		Amyloxenasma	0.013	0.003
		unidentified	0.004	0.000
	Atheliales	*Athelia*	0.010	0.002
		Leptosporomyces	0.160	0.014
		Piloderma	0.004	0.000
		Tylospora	0.017	0.014
		unidentified	0.000	0.005
	Auriculariales	*Elmerina*	0.012	0.000
		Exidia	0.009	0.012
		unidentified	0.003	0.014
	Boletales	*Boletus*	0.211	0.022
		Coniophora	0.013	0.001
		Gyrodon	0.007	0.000
		Hydnomerulius	0.012	0.001
		Imleria	0.064	0.018
		Leucogyrophana	0.005	0.002
		Paxillus	0.401	0.016
		Pseudomerulius	0.021	0.000
		Serpula	0.109	0.017
		Suillus	0.265	0.009
		Tapinella	0.103	0.020
		Xerocomellus	0.022	0.000
		Xerocomus	0.013	0.007
		unidentified	0.012	0.001
	Cantharellales	*Botryobasidium*	0.392	0.093
		unidentified	0.034	0.000
	Cantharellales	*Ceratobasidium*	0.017	0.005
		Sistotrema	0.133	0.027
		Thanatephorus	0.003	0.018
		unidentified	0.266	0.265
	Corticiales	*Cinereomyces*	0.020	0.022
		Crustoderma	0.022	0.000
		Cylindrobasidium	0.005	0.007
		Hyphodontia	0.304	0.118
		Hypochnicium	0.021	0.003
		Phlebia	0.887	0.168
		Schizopora	0.007	0.000

		Scopuloides	0.202	0.044
		Vuilleminia	0.012	0.003
		unidentified	0.008	0.069
	Gloeophyllales	*Gloeophyllum*	0.003	0.005
	Hymenochaetales	*Fomitiporella*	0.003	0.000
		Fuscoporia	0.042	0.021
		Inonotus	0.018	0.000
		Odonticium	0.003	0.000
		Peniophorella	0.116	0.061
		Phaeolus	0.003	0.000
		Phellinus	0.087	0.022
		Pseudochaete	0.003	0.000
		Resinicium	0.330	0.033
		Tubulicrinis	0.007	0.000
		Xanthoporia	0.044	0.002
		unidentified	0.026	0.013
	Phallales	*Phallus*	0.000	0.123
	Polyporales	*Abortiporus*	0.055	0.000
		Alutaceodontia	0.031	0.000
		Antrodia	0.183	0.048
		Antrodiella	0.061	0.017
		Aphanobasidium	0.199	0.025
		Atheliachaete	0.000	0.003
		Aurantiporus	0.775	0.036
		Bjerkandera	1.000	0.376
		Burgoa	0.007	0.000
		Byssomerulius	0.000	0.025
		Cabalodontia	0.008	0.000
		Ceraceomyces	0.021	0.021
		Ceriporia	0.535	0.242
		Ceriporiopsis	0.043	0.005
		Chondrostereum	0.026	0.001
		Coriolopsis	0.000	0.007
		Daedaleopsis	0.023	0.005
		Datronia	0.027	0.015
		Fibroporia	0.284	0.014
		Fomes	0.039	0.070
		Fomitopsis	0.111	0.015
		Funalia	0.077	0.076
		Ganoderma	0.029	0.031
		Gloeoporus	0.007	0.029
		Hapalopilus	0.021	0.045
		Hyphoderma	0.042	0.040
		Hyphodermella	0.000	0.012
		Irpex	0.000	0.028
		Junghuhnia	0.185	0.020
		Laetiporus	0.116	0.032
		Lentinus	0.025	0.020
		Lenzites	0.009	0.001
		Marchandiomyces	0.000	0.005
		Mycoacia	0.180	0.028

		Panus	0.000	0.006
		Phanerochaete	0.072	0.100
		Phlebiopsis	0.033	0.032
		Pilatoporus	0.031	0.008
		Piptoporus	0.700	0.025
		Polyporales	0.008	0.000
		Polyporus	0.009	0.013
		Postia	0.384	0.032
		Rhodonia	0.000	0.003
		Rigidoporus	0.038	0.000
		Sarcodontia	0.044	0.000
		Sarcoporia	0.009	0.000
		Schizophyllum	0.016	0.379
		Skeletocutis	0.174	0.023
		Sparassis	0.005	0.000
		Steccherinum	0.358	0.219
		Trametes	0.272	0.270
		Trichaptum	0.025	0.006
		Tyromyces	0.803	0.066
		Uncobasidium	0.146	0.001
		Xenasma	0.003	0.000
		Xylodon	0.003	0.012
		unidentified	3.151	2.021
	Russulales	*Artomyces*	0.055	0.003
		Asterostroma	0.012	0.000
		Auriscalpium	0.039	0.036
		Gloeocystidiellum	0.016	0.008
		Gloiothele	0.005	0.000
		Hericium	0.000	0.005
		Lactarius	0.034	0.000
		Laxitextum	0.005	0.012
		Peniophora	0.001	0.017
		Russula	0.017	0.006
		Scytinostroma	0.014	0.003
		Stereum	0.144	0.175
		unidentified	3.363	2.175
	Sebacinales	unidentified	0.000	0.009
	Thelephorales	*Brevicellicium*	0.009	0.000
		Luellia	0.008	0.000
		Pseudotomentella	0.009	0.000
		Sistotremastrum	0.039	0.017
		Tomentella	0.010	0.007
		Trechispora	0.480	0.137
		unidentified	0.034	0.009
	Agaricostilbales	*Bensingtonia*	0.232	0.017
		Kondoa	0.081	0.053
		Kurtzmanomyces	0.013	0.000
		Ruinenia	0.008	0.000
		Sterigmatomyces	0.000	0.003
	Cystobasidiales	*Buckleyzyma*	0.017	0.101
		Cystobasidium	0.005	3.000

		Symmetrospora	0.001	0.067
	Erythrobasidiales	*Bannoa*	0.027	0.021
		Erythrobasidium	0.010	0.067
		Sakaguchia	0.000	0.005
	Entylomatales	*Entyloma*	0.014	0.000
		Tilletiopsis	0.243	0.028
		unidentified	0.436	0.432
	Exobasidiales	*Acaromyces*	0.000	0.002
		Exobasidium	0.001	0.018
	Golubeviales	*Golubevia*	0.029	0.012
	Tilletiales	*Tilletia*	0.004	0.000
		unidentified	0.009	0.000
	Malasseziales	*Malassezia*	1.000	0.093
		unidentified	0.014	0.000
	Microbotryales	*Microbotryum*	0.038	0.009
		Sampaiozyma	0.001	0.006
	Sporidiobolales	*Rhodosporidiobolus*	0.029	0.000
		Rhodotorula	0.008	1.000
		Sporobolomyces	4.000	1.000
		unidentified	0.001	0.002
	Pucciniales	*Melampsora*	0.036	0.006
		Pucciniastrum	0.004	0.000
	Cystofilobasidiales	*Cystofilobasidium*	0.013	0.015
		Guehomyces	0.004	0.014
		Mrakiella	0.010	0.000
		Itersonilia	0.172	0.209
		Mrakia	0.000	0.002
		Udeniomyces	3.000	0.122
		unidentified	0.000	0.007
	Filobasidiale	*Filobasidium*	0.047	0.197
		Naganishia	0.001	0.008
		Solicoccozyma	0.021	0.002
	Holtermanniales	*Holtermanniella*	0.003	0.016
	Tremellales	*Bullera*	0.332	0.069
		Bulleribasidium	0.000	0.001
		Cryptococcus	0.008	3.000
		Dioszegia	0.061	0.229
		Fellomyces	0.000	0.012
		Genolevuria	0.020	0.017
		Hannaella	0.035	0.006
		Papiliotrema	0.009	0.134
		Sirobasidium	0.000	0.003
		Tremella	0.000	0.002
		Vishniacozyma	0.051	0.264
		unidentified	0.005	0.024
	Trichosporonales	*Apiotrichum*	0.009	0.109
		Cutaneotrichosporon	0.017	0.009
	Urocystidales	*Urocystis*	0.016	0.020
	Ustilaginales	*Anthracocystis*	0.008	0.000
		Kalmanozyma	0.009	0.005
		Melanopsichium	0.004	0.000

		Moesziomyces	0.000	0.007
		Sporisorium	0.021	0.000
		Tranzscheliella	0.009	0.016
		Ustilago	0.721	0.497
		unidentified	0.535	0.075
	Wallemiales	*Wallemia*	0.206	0.315
Chytridiomycota	*Rhizophlyctidales*	*Rhizophlyctis*	0.000	0.005
	Spizellomycetales	*Powellomyces*	0.000	0.006
Entomophthoromycota	*Basidiobolales*	*Basidiobolus*	0.000	0.008
	Entomophthorales	*Conidiobolus*	0.161	0.000
Mortierellomycota	*Mortierellales*	*Mortierella*	0.059	8.000
Mucormycota	*Mucorales*	*Mucor*	0.000	0.016
	Mucorales	*Rhizopus*	0.001	0.000
	unasignated		6.000	8.000
	unidentified		3.927	5.676

*percentage share of studied genera greater than 0.001%; ** Kingdom Protista

Table S3. The limits of detection of extrolites in dust samples.

Metabolite	LOD (µg/kg)
15-Acetyldeoxynivalenol	4.7
15-Hydroxyculmorin	1.7
15-Methyl_*epi*_Fumiquinazolin A	N/A
16-Ketoaspergillimide	0.13
2-Chlorunguinol	0.02
2-Methylmitorubin	1.4
3-Acetyldeoxynivalenol	4.8
3-Nitropropionic acid	0.71
4-Hydroxyalternariol	2.2
4-Monoacetoxyscirpenol	N/A
5-Methylmellein	2.6
7-Hydroxykaurenolid	0.67
7-Hydroxypestalotin	0.49
A 23187	N/A
AAL-TA Toxin	1.5
Abscisic acid	15
Acetylchaetoglobosin D	0.65
Acuminatum B	7.3
Acuminatum C	17
Aflatoxicol	0.23
Aflatoxin B1	0.17
Aflatoxin B2	0.04
Aflatoxin G1	0.10
Aflatoxin G2	0.35
Aflatoxin M1	0.11
Aflatoxin M2	N/A
Aflatoxin P1	0.11
Aflatoxin Q1	0.50
Aflatrem	N/A
Aflavarin	N/A
Agistatin B	248
Agistatin D	6.3
Agistatin E	2.1
Agroclavin	0.10
Aigualomycin D	2.6
Alamethicin F30	0.45
alpha Zearalenol	0.59
alpha Zearalenol Glucoside	1.5
Alteichin	N/A
Altenusin	29
Alternarian acid	127
Alternariol	0.10
Alternariol-3-Glucoside	1.1
Alternariol-9-Glucoside	0.87
Alternariolmethylether	0.11
Alternariolmethylether-glucoside	10
Altersetin	0.89
Altersolanol	126

Altertoxin II	N/A
Altertoxin-I	0.96
Amidepsin B	0.97
Aminodimethyloctadecanol	13
Amoxycillin	N/A
Amphotericin	N/A
Anacin	0.93
Andrastin A	0.22
Andrastin B	0.83
Andrastin C	N/A
Andrastin D	N/A
Anisomycin	0.25
Anomalin A	N/A
Antibiotic F1849 A	0.22
Antibiotic L 696474	27
Antibiotic PF 1052	2.5
Antibiotic Y	6.9
Apicidin	0.20
Ascochlorin	0.07
Ascofuranone	0.07
Ascomycin	1.1
Asparason A	N/A
Aspercolorin	1.1
Asperfuran	3.7
Aspergamid A	0.65
Aspergillicin Derivative	0.30
Aspergillimide	0.03
Aspergillin PZ	N/A
Asperglaucide	0.03
Asperlacton	0.66
Asperloxine A	0.26
Asperphenamate	0.39
Asperthecin	N/A
Aspinolid B	0.07
Aspinonene	0.35
Aspinonene	N/A
Aspochalasin C	5.3
Aspochalasin D	0.39
Aspochalasin I	1.2
Aspochalasin J	1.7
Aspterric acid	18
Aspyrone	5.3
Asteltoxin	0.68
Asterric acid	0.15
Atlantinon A	0.65
Atpenin A5	0.21
Atroventinmethylether	0.27
Aurantiamin A	0.59
Auranticin A	5.2
Aurantin	0.26
Aurasperon B	N/A

Aurasperon C	N/A
Aurasperon G	N/A
Aureobasidin A	2.9
Aurofusarin	1.1
Austalide A	0.57
Austalide B	0.11
Austalide D	0.34
Austalide F	0.65
Austamide	0.58
Austdiol	N/A
Austocystin A	0.11
Austocystin D	0.81
Austocystin I	0.55
Averantin	0.04
Averantinmethylether	0.03
Averufin	0.02
Averufin Derivat	0.02
Bacitracin	N/A
Bafilomycin A1	0.27
Barceloneic acid	0.55
Bassianolide	0.02
Beauvericin	0.06
Benzomalvin A	2.1
Benzomalvin C	0.36
Berkedrimane B	0.27
Berkeleyacetal B	5.6
beta Zearalenol	2.9
beta-Zearalenol Glucosid	0.85
Bikaverin	0.46
Bismethylthiogliotoxin	0.86
Brasiliamide A	0.27
Brefeldin A	20
Brevianamid F	0.35
Brevicompanine B	0.08
Butenolid	3.2
Butyrolacton II	2.8
Butyrolacton III	0.52
Butyrolactone I	1.1
Calonectrin	19
Calphostin C	3.4
Calyxanthone	3.9
Carnequinazolin A	0.24
Cephalochromin	N/A
Cephalosporin C	N/A
Cercosporamide	0.09
Cercosporin	11
Cereulide	0.19
Chaetocin	38
Chaetoglobosin D	30
Chaetoviridin A	0.88
Chanoclavin	0.02

Chetomin	1.0
Chetoseminudin	N/A
Chevalone C	0.25
Chlamydospordiol	0.08
Chlamydosporol	0.34
Chloramphenicol	0.03
Chlorocitreorosein	0.63
Chloronectrin.2	0.06
Chlortetracyclin	N/A
Chrodrimanin	9.1
Chromomycin A3	N/A
Chrysogin	0.56
Chrysophanol	4.0
Cinereanin	1.3
Citreohybriddione	19
Citreohybridinol	0.09
Citreorosein	0.74
Citreoviridin	0.18
Citreoviridin C	N/A
Citreoviridinol	N/A
Citrinin	0.30
Cladosporin	1.1
Clonostachydiol	0.33
CNM 115443	0.43
Cochliodinol	N/A
Cochlioquinone A	N/A
Colchicin	0.51
Communesin B	0.27
Culmorin	1.3
Curvularin	0.36
Curvulin	0.27
cyclo(L-Leu-L-Pro)	0.53
cyclo(L-Pro-L-Tyr)	8.5
cyclo(L-Pro-L-Val)	1.2
Cycloaspeptide A	0.09
Cycloechinulin	0.11
Cycloheximide	3.6
Cyclopenin	0.09
Cyclopenol	0.82
Cyclopeptine	0.05
Cyclosporin A	5.4
Cyclosporin B	1.1
Cyclosporin C	9.2
Cyclosporin D	2.4
Cyclosporin H	0.83
Cylindrocarpon A4	0.01
Cylindrol B	0.03
Cytochalasin A	1.7
Cytochalasin B	1.5
Cytochalasin C	0.69
Cytochalasin D	0.16

Cytochalasin E	0.43
Cytochalasin H	2.0
Cytochalasin J	1.2
Daunorubicin	21
Deacetylneosolaniol	1.3
Decalonectrin	2.6
Decarestrictin	28
Dechlorogriseofulvin	0.43
Dechloroisochromophilon IV	1.2
Dechoronornidulin	0.08
Deepoxy-DON	7.8
Dehydroaustinol	1.1
Dehydrocurvularin	0.96
Dehydrocyclopeptine	1.1
Dehydrogriseofulvin	0.03
Demethylasteltoxin	0.87
Demethylsulochrin	0.58
Deoxyaltersolaniol A	N/A
Deoxyaltersolanol	11
Deoxybrevianamid E	2.1
Deoxyfusapyron	0.80
Deoxynivalenol	4.0
Deoxynivalenol-15-Sulfate	N/A
Deoxynivalenol-3-Sulfate	N/A
Deoxynortryptoquivalin A	0.62
Deoxypentahydroxyscirpenol	N/A
Deoxytryptoquialanine	N/A
Deoxytryptoquivaline A	0.27
Desoxypaxillin	N/A
Desoxyverrucosidin	N/A
Destruxin A	0.07
Destruxin B	0.33
Destruxin CHL	0.79
Destruxin D	0.37
Destruxin-Ed Derivat	0.66
Dethiosecoemestrin	N/A
Diacetoxyscirpenol	0.14
Dichlordiaportin	0.64
Dichlormethylasterric acid	0.36
Dihydrobotrydial	N/A
Dihydrochlamydocin	0.22
Dihydrocitrinone	0.77
Dihydroergosine	0.08
Dihydroergotamin	0.24
Dihydrogriseofulvin	0.95
Dihydrogriseofulvin	N/A
Dihydroinfectopyron	0.32
Dihydrosterigmatocystin	N/A
Dihydrotrichotetronine	2.3
Dihydroxycalonectrin	11
Dihydroxycalonectrin	N/A

Dihydroxymellein	0.51
DihydroxyZONdimethylether	2.6
Dihydroxy-ZON-Mrethylether	N/A
Dimethoxymethylgrisantrion	N/A
Dinactin	N/A
Diplodiatoxin	2.0
Diydrolysergol	0.10
DON-3-Glucoside	7.6
DON-Glutathion	N/A
Doxorubicin	11
Doxycyclin	N/A
Drimane 6	0.48
Drimane 8	1.1
Elymoclavin	0.18
Elymoclavin-Fructoside	0.15
Emindole SA	0.61
Emindole SA	N/A
Emodin	0.06
Endocrocin	15
Enniatin A	0.007
Enniatin A1	0.06
Enniatin B	0.006
Enniatin B1	0.03
Enniatin B2	0.17
Enniatin B3	0.001
Epiequisetin	0.10
Epoxyagroclavin	0.21
Epoxyagroclavin	N/A
Epoxycytochalasin C	0.84
Equisetin	0.10
Eremofortin A	0.77
Ergine	0.04
Ergocornine	0.36
Ergocorninin	0.19
Ergocristam	N/A
Ergocristinam	N/A
Ergocristine	0.35
Ergocristinin	0.12
Ergocryptin	0.28
Ergocryptinin	0.18
Ergometrin	0.71
Ergometrinin	0.03
Ergosin	0.42
Ergosinin	0.04
Ergotamin	0.47
Ergotaminin	0.23
Ergovaline	N/A
Erythromycin	N/A
F01-1358A	0.22
Fallacinol	0.11
Fellutannine	0.48

Fellutannine B	1.9
Festuclavin	0.02
FK 506	1.0
FK 9775 A	N/A
FK 9775 B	N/A
Flavipucin	0.59
Flavoglaucin	0.03
Folipastin	0.04
Fonsecin	N/A
FS4	N/A
Fulvic acid	13
Fumagillin	9.0
Fumagillol	10
Fumigaclavin A	0.12
Fumigaclavin C	0.83
Fumiquinazolin A	0.18
Fumiquinazolin D	0.27
Fumiquinazolin Derivat	0.85
Fumiquinazolin E	N/A
Fumiquinazolin F	11
Fumiquinazolin I	N/A
Fumitremorgin A	0.89
Fumitremorgin B	N/A
Fumitremorgin C	0.19
Fumonisin A1	N/A
Fumonisin A1 Vorstufe	0.59
Fumonisin A2	N/A
Fumonisin B1	2.4
Fumonisin B2	1.7
Fumonisin B3	5.8
Fumonisin B4	N/A
Fumonisin B6	N/A
Fungerin	0.20
Fusaproliferin	40
Fusapyron	0.73
Fusarenon-X	3.9
Fusarielin A	39
Fusarin C	42
Fusarinolic acid	N/A
Geldanamycin	0.46
Geodin	17
Geodin hydrate	0.40
Gibberellic acid	2.2
Gibepyron D	4.3
Gliocladic acid	2.4
Gliotoxin	0.91
Glyantrypin	0.85
Gregatin B	N/A
Griseofulvin	0.14
Griseophenone B	1.1
Griseophenone C	0.33

Harzianopyridine	0.07
Harzianum A	13
HC-Toxin	1.9
Helvolic acid	2.1
Helvolinic acid	12
Helvolinic acid	N/A
Heptaibin	24
Heptelidic acid	8.7
Herquline A	0.06
HT-2 Glucoside	1.7
HT-2 Toxin	0.81
hydrolysed Fumonisin B1	0.20
hydrolysed Nidulin	N/A
Hydroxycarnequinazolin A	0.87
Hydroxycurvularin	0.94
Hydroxyroquefortine C	N/A
Hydroxysydonic acid	1.8
Hypothemycin	3.7
Ilicicolin A	0.02
Ilicicolin B	0.58
Ilicicolin C	0.23
Ilicicolin E	0.03
Infectopyron	13
Integracin A	0.04
Integracin B	0.09
Ionomycin	8.1
Irgasan	129
Isochromophilon III	22
Isochromophilon IV	2.0
Isochromophilon IX	0.19
Isochromophilone VI	0.80
Isofusidienol	0.07
Isokotanin B	0.24
Iso-Rhodoptilometrin	0.03
Josamycin	N/A
K 252a	0.38
K252b	7.3
K-76 Derivative 4	0.52
Kipukasin B	0.28
Kipukasin D	0.94
KO 143	7.5
Kojic acid	20
Koninginin D	2.6
Koninginin E	0.63
Linamarin	2.3
Lincomycin	N/A
LL-Z 1272e	0.02
Lolitrem B	N/A
Lolitrem N	N/A
Lotaustralin	1.3
Luteoskyrin	N/A

Luteusin A	0.71
Lysergol	0.24
Macrosphaelide A	0.20
Macrosporin	0.13
Malformin A	0.10
Malformin C	0.11
Marcfortine A	0.04
Marcfortine B	N/A
Marcfortine C	N/A
Meleagrin	0.54
Meleagrin Derivative	0.27
Methoxycurvularin	0.45
Methoxysterigmatocystin	0.03
Methylasterric acid	0.12
Methylequisetin	0.08
Methylfunicon	0.04
Methylsulochrin	0.03
Methysergide	0.04
Mevastatin	1.4
Mevinolin	0.21
Mithramycin C	N/A
Mitomycin	N/A
Monactin	0.15
Moniliformin	1.0
Monoacetoxyscirpenol	1.2
Monocerin	0.06
Monomethylcurvulin	1.3
Mycophenolic acid	1.1
Mycophenolic acid IV	0.20
Myriocin	2.7
Mytoxin C	26
N-Benzoylphenylalanin	0.13
Neoechinulin A	0.94
Neosolaniol	0.15
Neoxaline	0.12
NG 012	1.4
Nidulin	0.01
Nigericin	0.03
NIgragillin	N/A
Nigrosporoate A	7.0
Nivalenol	0.77
Nivalenol Glucoside	1.1
Nonactin	0.05
Norlichexanthone	0.23
Nornidulin	0.03
Norsolorinic acid	0.03
Nortryptoquialanine	N/A
Norverrucosidin	0.57
Notoamide Derivative	0.10
Notoamide E Derivative	0.10
NP 12318	0.08

NP 19199	4.5
NP 8140	17
NT-2 Toxin	0.36
NX-1	N/A
NX-2	N/A
NX-3	N/A
Ochratoxin A	0.14
Ochratoxin alpha	0.95
Ochratoxin B	0.11
Ochratoxin C	0.01
Ochrephilone	0.08
Okaramine B	0.28
Okaramine D	0.88
Oligmycin B	14
Oligomycin A	7.9
O-Methylsterigmatocystin	0.04
O-Methylviridicatin	0.02
Ophiobolin A	4.4
Ophiobolin B	32
Oxalicine B	2.2
Oxaline	0.08
oxidized Elymoclavin	2.3
oxidized Luol	0.46
Oxytetracyclin	N/A
Papyracillic acid A	4.0
Paracelsin A	N/A
Paracelsin B	N/A
Paraherquamide A	0.09
Paraherquamide E	0.36
Paspalic acid	5.4
Paspalin	N/A
Paspalinin	N/A
Paspalitrem A	N/A
Paspalitrem B	N/A
Patulin	11
Paxillin	3.7
Penicillazaphilone B	0.61
Penicillic acid	0.45
Penicillide	0.97
Penicillin V	N/A
Penigequinolone A	0.12
Penitrem A	0.41
Pennigritrem A	2.3
Pentoxyfylline	0.04
Pestalotin	0.64
Phenopyrrozin	0.31
Phomalactone	0.61
Phomalone	0.06
Phomopsin A	3.8
Phomopsin B	N/A
Phomopsolide B	1.1

Physcion	6.8
Pinselin	0.60
Piscarinin A	0.46
Porritoxinol	1.2
PR Toxin	N/A
Prehelminthosporol	N/A
Prehelminthosporollacton	N/A
Prelaptin	0.31
Pseurotin A	1.5
Pseurotin D	N/A
Puberulin A	0.66
Puromycin	0.29
Purpactin A	0.33
Purpuride	0.08
Pyranonigrin	5.6
Pyrenocin A	1.6
Pyrenophorol	0.96
Pyripyropene A	0.58
Pyripyropene B	N/A
Pyripyropene D	0.29
Pyrophen	0.26
Quadrone	2.3
Questiomycin A	0.82
Quinadoline A	0.74
Quinadoline B	2.7
Quinolactacin A	0.009
Quinolone A	2.1
Radicicol	0.19
Radiclonic acid	23
Rapamycin	15
Rasfonin	3.1
Roquefortine C	0.35
Roquefortine D	0.48
Roquefortine E	0.08
Roridin A	1.0
Roridin L-2	N/A
Rorotoxin A	16
Rubellin D	0.09
Rubratoxin A	N/A
Rugulosin	1.2
Rugulotrosin	24
Rugulovasine A	0.12
Rugulusovin	0.45
Sambucinol	4.5
Satratoxin F	N/A
Satratoxin G	N/A
Satratoxin H	N/A
Scalusamid A	0.32
Sch 725680	N/A
Sclerotioramin	0.30
Sclerotiorin	3.7

Secalonic acid D	0.86
seco-Sterigmatocystin	0.13
semi Xanthomegnin	N/A
semi-Xanthomegnin	1.5
Setosusin	1.1
Siccanin	0.93
Siccanol	N/A
Skyrin	0.08
S-Methyl-deoxynivalenol	N/A
Sphingofungin D	0.77
Spiramycin	N/A
Spirodihydrobenzofuranlactam IV	0.46
Sporogen AO I	3.1
Stachybotryamide	0.69
Stachybotrylactam	0.22
Sterigmatocystin	0.06
Sulochrin	0.16
Sydonic acid	0.08
Sydowinin A	2.9
Synazerol	N/A
T-2 Toxin	0.41
T2-Glucoside	N/A
T2-Triol	2.6
Taxol	N/A
Tensidol B	N/A
Tentoxin	0.11
Tenuazonic acid	30
Ternatin	0.27
Terpendole C	N/A
Terpendole E	N/A
Terphenyllin	2.1
Terrecyclic acid	4.0
Terretonin	3.4
Terretonin F Derivat	1.4
Territrem B	0.59
Tetrahydrobostrycin	1.1
Thielavin B	0.29
Thiolutin	3.5
TR-2 Toxin	N/A
Triacetyl-DON	1.8
Trichalasin B	0.34
Trichodermamide C	0.85
Trichodermin	1.2
Trichodimerol	0.27
Trichostatin A	0.30
Trichotetronin	2.2
Trichothecin	N/A
Trichothecolone	N/A
Trypacidin	0.09
Trypacidin	N/A
Tryprostatin A	0.41

Tryprostatin B	1.5
Tryprostatin B	N/A
Tryptophol	3.5
Tryptoquialanine	N/A
Tryptoquialanine Derivat	0.39
Tryptoquialanone	N/A
Tryptoquivaline A	0.48
Tryptoquivaline F	0.67
Tryptoquivaline G	0.32
Tylosin	N/A
Unguinol	0.06
Unugisin E	0.75
Usnic acid	0.02
Ustiloxin A	N/A
Ustiloxin B	N/A
Ustiloxin D	N/A
Ustusol A	0.17
Valinomycin	0.47
Vancomycin	N/A
Vermistatin	0.24
Verrucarin A	1.2
Verrucarin A	N/A
Verrucarin J	5.6
Verrucarin J	N/A
Verrucarol	N/A
Verrucofortine	0.02
Verrucosidin	N/A
Verruculogen	4.6
Verruculotoxin	0.08
Versicolorin A.2	0.05
Versicolorin C	0.13
Verticillin A	0.92
Violaceic acid	1.6
Viomellein	22
Vioxanthin	N/A
Viridicatin	0.15
Viridicatol	2.9
Viridicatumtoxin	87
Viridol	2.6
WIN 64821	0.33
WIN 68577	3.6
Wortmannin	1.3
Xanthomegnin	43
Xanthotoxin	0.04
Yaequinolone J2	N/A
Zaragozic acid A	N/A
Zearalenone	0.16
Zearalenone-16-Glucoside	N/A
Zearalenone-4-Glucoside	N/A
Zearalenone-14-Sulfate	N/A
Zinndiol	2.6

Zinniamide	1.1
Zinniol	5.1

N/A- no quantitative standard available.

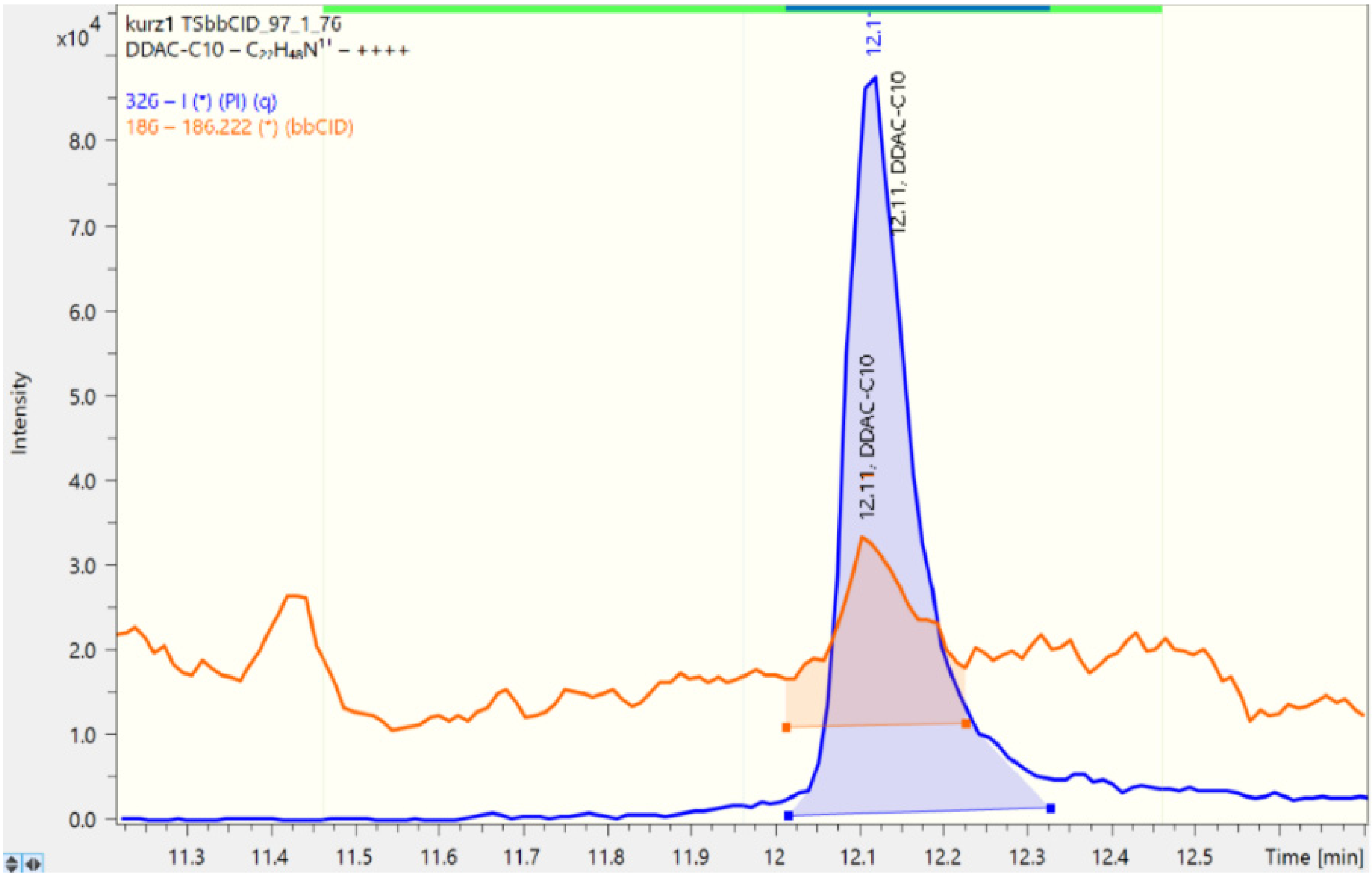

Figure S1. EICs of DDAC-C10 ions measured with bbCID Targetscreener positive method.
